# DExD-box helicase 39 A, targeted by coumestrol, facilitates the malignant behaviors of osteosarcoma cells

**DOI:** 10.1186/s41065-025-00588-0

**Published:** 2025-10-28

**Authors:** Wang Haiqing, Jin Yangli, Zhang Feng

**Affiliations:** 1 Department of Foot and Ankle Surgery, Ningbo No.6 Hospital, Ningbo, Zhejiang 315040 China; 2 Ningbo Clinical Research Center for Orthopedics, Sports Medicine & Rehabilitation,, Ningbo, Zhejiang 315040 China; 3 Department of Ultrasound, Ningbo Yinzhou No.2 Hospital, Zhejiang 315100 Ningbo, China

**Keywords:** Osteosarcoma, DDX39A, Prognosis, Cell cycle, Coumerstrol.

## Abstract

**Background:**

Osteosarcoma (OS) has a high degree of malignancy, is prone to metastasize. This study aims to identify new biomarkers and natural drugs for OS.

**Methods:**

Open access OS-related datasets in Gene Expression Omnibus database (GSE39262, GSE21257 and GSE16088) were retrieved and analyzed. The genes related to OS progression were screened through differentially expressed gene analysis, weighted gene co-expression network analysis and univariate COX regression analysis. Immune infiltration analysis was performed using the CIBERSORT and ESTIMATE methods. The binding relationship between DExD-box helicase 39 A (DDX39A) and the natural drug coumerstrol was verified with molecular docking and cellular thermal shift assay. OS cell lines with *DDX39A* knockdown and overexpression were constructed respectively, to investigate the functions of DDX39A and coumerstrol. Cell viability, proliferation, apoptosis, cell cycle distribution, migration and invasion abilities were analyzed by CCK-8, EdU, flow cytometry and Transwell assays, respectively.

**Results:**

*DDX39A* was highly expressed in OS samples and cell lines, associated with the poor prognosis of the patients. In vitro experiments confirmed that knockdown of *DDX39A* could inhibit the viability, proliferation, migration and invasion ability of OS cells, causing G1/S phase arrest and apoptosis. DDX39A had a good binding ability with coumerstrol. Coumerstrol treatment could inhibit the expression of *DDX39A* in OS cells and repressed the malignant behaviors of OS cells, while the overexpression of *DDX39A* reversed this phenomenon.

**Conclusion:**

DDX39A is a promising biomarker and therapy target for OS, and coumestrol exerts tumor suppressive properties in OS via inhibiting *DDX39A*.

**Supplementary Information:**

The online version contains supplementary material available at 10.1186/s41065-025-00588-0.

## Introduction

Osteosarcoma (OS) is a common primary bone tumor that originates from primitive mesenchymal cells [1]. OS is more common in children and adolescents [2]. The poor prognosis of OS is mainly due to metastasis, especially lung metastasis [3, 4]. At present, surgical resection, radiotherapy and multi-drug systemic therapy constitute the treatment strategies in clinical practice [5]. The 5-year survival rate of patients with localized OS remains at 60–70% [6]. However, with metastasis, the 5-year survival rate of patients was less than 30% [7, 8]. At present, there is no successful targeted therapy for OS with convincing clinical data [6, 9]. It is urgent to explore new targets and therapeutic drugs, in order to improve the prognosis of OS patients.

At present, bioinformatics has been widely applied to clarify the biological mechanisms of the pathogenesis and development of various human diseases, which is conducive to exploring new disease-related biomarkers and therapeutic targets [10, 11]. Specifically, the prognostic value of ferroptosis-related genes in the risk stratification of OS patients was evaluated by constructing a risk model, and two ferroptos-related therapeutic targets, hypoxia inducible lipid droplet associated (HILPDA) and mucin 1, cell surface associated (MUC1) were identified [12]. Weighted gene co-expression network analysis (WGCNA) is a bioinformatics method capable of systematically analyzing the gene expression patterns of multiple samples. It is capable of integrating highly relevant genes into multiple modules and analyzing the relationship between the modules and specific characteristics/traits (for example, the clinical information of patients) [13]. A recent study identifies charged multivesicular body protein 4 C (CHMP4C) as a biomarker and therapy target significantly associated with the prognosis of OS based on WGCNA [14]. However, the molecular mechanisms of the occurrence and development of OS are far from been fully clarified.

In this study, OS-related bulk transcriptome datasets (GSE39262 and GSE21257) from the Gene Expression Omnibus (GEO) database were analyzed, and the genes related to the clinical characteristics of OS were identified through differentially expressed genes (DEGs) analysis and WGCNA, and verified with the external dataset GSE16088. DExD-box helicase 39 A (DDX39A) was identified as a potential drug target in OS, and molecular docking techniques indicated that coumestrol was a candidate natural drug targeting DDX39A, which was validated by in vitro assays.

## Methods and materials

### Datasets

From Gene Expression Omnibus (GEO) database (https://www.ncbi.nlm.nih.gov/geo/), GSE39262, GSE21257 and GSE16088 datasets were downloaded. GSE39262 includes gene expression profile data of 5 primary untransformed cell line samples and 46 OS cell line samples. Screening of DEGs was conducted using the limma method, with the threshold set at |log fold change(FC)|≥ 0.58, adjusted *P* value < 0.05. GSE21257 includes 34 metastatic samples and 19 non-metastatic tissue samples for univariate COX survival analysis. GSE16088 dataset includes 6 normal samples and 14 OS tissue samples.

### WGCNA

WGCNA was performed using the Sangerbox platform [15] to identify the gene modules highly related to the occurrence and metastasis of OS. The median absolute deviation (MAD) of each gene was calculated, and the top 50% of the genes with the smallest MAD were eliminated. Subsequently, the “goodSamplesGene” function was used to perform sample clustering to identify and delete the outliers. The “pickSoftThreshold” function was used to calculate and determine the soft threshold. The dynamic tree cutting method was used to identify different gene modules, and the minimum number of genes in each module was 30. The sensitivity was set to 3, and mergeCutHeight was set to 0.25, and the modules were merged. The grey module was regarded as a collection of genes that could not be assigned to any module. The correlation between the module genes and clinical information was further analyzed. Meanwhile, the correlation between the traits and the genes were evaluated with gene significance (GS) and module membership (MM). The module with the highest correlation (|correlation coefficient| >0.5, *P* value < 0.05) was regarded as the key module, and all the genes in the module were considered as the core genes.

### Functional enrichment analysis

The Database for Annotation, Visualization and Integrated Discovery (DAVID) (https://david.ncifcrf.gov/summary.jsp) and the Metascape database (https://metascape.org/gp/index.html#/main/step1) were applied for gene ontology (GO) analysis [including three aspects: biological processes (BP), cellular components (CC), and molecular functions (MF)], Reactome pathway enrichment analysis, and Kyoto Encyclopedia of Genes and Genomes (KEGG) pathway enrichment analysis.

### Screening and functional analysis of core genes

With the Linkedomics database (https://www.linkedomics.org/login.php) and the data in TCGA-SRAC cohort from the UCSC Xena database (https://xenabrowser.net/datapages/), the co-expression network of DDX39A was analyzed. Subsequently, enrichment analyses of GO (biological processes) and KEGG pathways of *DDX39A* were conducted with gene set enrichment analysis (GSEA) with GSEA software (version 3.0) (http://software.broadinstitute.org/gsea/index.jsp). The samples were divided into the high-expression group (> = 50%) and the low-expression group (< 50%) based on the expression level of *DDX39A*, and the reference gene sets (c2.cp.kegg.v7.4.symbols.gmt and c5.go.bp.v7.4.symbols.gmt) were obtained from the Molecular Signatures Database (http://www.gsea-msigdb.org/gsea/downloads.jsp). The minimum gene set was set at “5”, and the maximum gene set was set at “5000”, and a thousand resampling times were conducted. *P* value < 0.05 and false discovery rate (FDR) < 0.25 were considered statistically significant.

### Immune infiltration analysis

“CIBERSORT” method was used to evaluate 22 types of immune cells in the samples of GSE39262 and GSE21257. The StromalScore, ImmuneScore and ESTIMATEscore of the samples were calculated using the “ESTIMATE” method.

### Cell culture

Human OS cell lines U-2 OS (HTB-96), 143B (CRL-8303), MG-63 (CRL-1427), HOS (CRL-1543), Saos-2 (HTB-85), and human osteoblast cell line hFOB1.19 (CRL-3602) (ATCC, Manassas, VA, USA) were certified by STR genotyping. All the cell lines were cultured in Dulbecco’s Modified Eagle Medium (DMEM) supplemented with 10% fetal bovine serum (FBS) and 1% penicillin and streptomycin (all from Gibco, Grand Island, NY, USA) in an incubator with 5% CO_2_ at 37 ° C. The Lipofectamine™ 3000 transfection kit (ThermoFisherScience, Waltham, MA, USA) was used to transfect control small interference RNA (siRNA) (NC), si-*DDX39A*#1, si-*DDX39A*#2 and si-*DDX39A*#3, *DDX39A* overexpression vector and empty vector into 143B and HOS cell lines. After transfection for 48 hours, the transfection efficiency was detected by quantitative reverse transcription-polymerase chain reaction (qRT-PCR) assay. The sequences for siRNAs: NC: 5’-UUCUCCGAACGUGUCCGGA-3’; si-*DDX39A*#1: 5’-GTGGAAAACGATCTTTTGGATTA-3’; si-*DDX39A*#2: 5’-AGGAATATGAGCGCTTTTCCAAG-3’ and si-*DDX39A*#3: 5’-AGCAGTACTACGTCAAACTCAAA-3’. Coumestrol [≥ 95.0% (HPLC), Sigma-Aldrich, Shanghai, China] was dissolved in dimethylsulfoxide (Sigma-Aldrich, Shanghai, China) and diluted by DMEM to different doses to treat the OS cells.

### qRT-PCR

After total RNA extraction with TRIzol (Invitrogen, Carlsbad, CA, USA), cDNA was obtained by reverse transcription using the PrimeScript RT kit (Takara, Dalian, China). Using the SYBR Green qPCR Master Mix kit (Thermo Fisher Scientific, Carlsbad, CA, USA), qRT-PCR was performed on the ABI PRISM^®^ 7300 system (ABI, Carlsbad, CA, USA). Using the 2^−△△Ct^ method, GAPDH was used as the endogenous control for quantifying the genes. All the primers were designed and synthesized by Bojie Biomedical Science and Technology Co., Ltd. (Wuhan, China). *DDX39A*: forward 5’-GCAGATTGAGCCTGTCAACG-3’ and reverse 5’-AGACCACCGAAGAACAC-3’; *SARNP*: forward 5’- GGGCAGCTAGGTTTGGGATT-3’ and reverse 5’- AAGCGCTCTGCTCTTTTCCT − 3’; DDX39B forward 5’-TCCAGGCCGTATCCTAGCC-3’ and reverse 5’-GCATGTCGAGCTGTTCAAGC-3’; *ALYREF*: forward 5’- GCAGGCCAAAACAACTTCCC-3’ and reverse 5’- AGTTCCTGAATATCGGCGTCT-3’; *THOC1*: forward 5’- TCTTCTGTGGACGGATTCAGC-3’ and reverse 5’- CTCGTCTCCCATTTCGCCTTC-3’; *THOC2*: forward 5’- GCCACCGGACTTAACCAAGA-3’ and reverse 5’- CTGTGCTTGTCCGAGGACTT-3’; *GAPDH*: forward 5’-CACTAGGCGCTCACTGTTCT-3’ and reverse 5’-CGCCCCACTTGATTTGGAG-3’.

### Cell viability assay

Cell viability was evaluated using a cell counting kit-8 (CCK-8) (Beyotime, Shanghai, China). The OS cells in different groups were inoculated into 96-well plates (5 × 10^3^ cells per well) for incubation for 1 day, 2 days, 3 days and 4 days. On each day, 10 µL of CCK-8 reagent was added into each well. Subsequently, the cells were cultured for additional 2 h, and then the absorbance at 450 nm was measured on a microplate reader (Bio-Rad, Carlsbad, CA, USA).

### 5-Ethynyl-2’-Deoxyuridine (EdU) assay

The OS cells on cover glasses in different groups were incubated with 50 µM EdU working solution [a mixture of the EdU kit (Beyotime, Shanghai, China) and the medium at a ratio of 1:1000] at room temperature for 2 h. After fixation with 4% paraformaldehyde for 30 min, the cells were immersed in glycine solution for 8 min. Next, the cells were incubated with 0.5% Triton X-100 in phosphate buffer saline (PBS). Then, in the dark, the OS cells were stained at room temperature with Apollo^®^ staining solution for 30 min, and then stained for 30 min with DAPI solution. Under the microscope, five fields of view were randomly selected, and the total number of cells stained with EdU (indicating proliferating cells) and the total number of cells stained with DAPI (indicating the total number of cells) were counted.

### Flow cytometry

To detect the apoptosis of OS cells, 100 µL of cell suspension (1 × 10^6^ cells/mL) was incubated with 5 µL of AnnexinV-FITC staining solution and 10 µL of propidium iodide (PI) staining solution, and incubated at room temperature in the dark for 15 min. Finally, the apoptosis of cells was analyzed using the FACS Calibur Flow Cytometer (BD Biosciences, SanJose, CA, USA). To detect the cell cycle distribution of the OS cells, the cells were washed with pre-cooled PBS, and fixed with 70% ethanol and stored at 4℃. The cells were resuspended with the binding buffer, and stained with PI in the dark for 30 min, and after washing, the cells were analyzed with the flow cytometer. Briefly, the gating strategy was based on the cell scatter plots [Forward SCatter-Area (FSC-A) vs. Side SCatter-Area (SSC-A)], to exclude the cell debris and instrument noise (the cells in the gate were defined as the P1 cells). Then in the P1 cells, the cells were further screened based on the cell scatter plots [Forward SCatter-Height (FSC-H) vs. FSC-A], to exclude the cell aggregates (the cells in the gate were defined as the P1 cells). Finally, the P2 cells were shown in a four-quadrant diagram based on the fluorescence intensity of FITC and PI (for apoptosis detection) or in a histogram based the on the fluorescence intensity of PI (for cell cycle detection).

### Transwell assay

Transwell chambers (with a pore size of 8 μm; Corning, Beijing, China) were used for cell migration and invasion experiments. For the invasion experiment, the matrigel (BD Biosciences, SanJose, CA, USA) was diluted with pre-cooled DMEM, and then 100 µL Matrigel was used to cover the filter. Matrigel was not used in the migration assays. The OS cells in different groups were resuspended in the medium without FBS. 100 µL of the cell suspension containing 1 × 10^5^ cells was added to the upper compartment, and 600 µL of complete medium was added to the lower compartment. The cells were cultured for 48 h. After fixation with 4% paraformaldehyde and staining with 5% crystal violet solution, the cells that passed through the filter membrane were finally observed and counted under a microscope (×200).

### Western blot

Sodium dodecyl sulfate-polyacrylamide gel electrophoresis was performed after total protein extraction, and the protein was transferred onto the polyvinylidene difluoride membrane (Millipore, Bedford, MA, USA). Subsequently, after blocking the membrane with 5% skimmed milk, the membrane was incubated overnight with primary antibodies at 4 °C. The primary antibodies were: anti-DDX39A (ab180857, 1:1000, Abcam, Shanghai, China) and anti-GAPDH (ab8245, 1:1000, Abcam, Shanghai, China). On the next day, the membrane was incubated with the secondary antibody Goat Anti-Rabbit IgG H&L (HRP) (ab6721, 1:2000, Abcam, Shanghai, China) at room temperature for 1 h. Finally, an enhanced chemiluminescence kit (Pierce, Waltham, MA, USA) was used for protein band development, and the signals were captured with the ChemiDoc Imaging System (Bio-Rad, Hercules, CA, China). The ImageJ software (National Institutes of Health, USA) was used to quantify the band intensity.

### Molecular docking

Potential drugs targeting DDX39A were retrieved from the HERB database (http://herb.ac.cn/). The 3D structures of small molecule drugs (coumestrol, PubChem CID: 5281707; quercetin PubChem CID: 5280343) were downloaded from the PubChem database and converted into Mol2 format using the Open Babel software. The 3D structure of DDX39A (PDB: 8IJU) was downloaded from the UniProt database. The small molecule drugs were hydrogenated using the AutoDocktools software, and the proteins were dehydrated, hydrogenated and charged. Finally, the pdbqt file was exported. Molecular docking was carried out on the AutoDock vina software. Briefly, the pdbqt files of the receptors (proteins) and the ligands (drugs) were imported, and then an appropriate docking boxes were set, and the config files were saved. Finally, 20 blind docking operations were performed, and the complex with the lowest binding energy was selected for visualization in the PyMoL software.

### Cellular thermal shift assay (CETSA)

OS cell lines were exposed to DMSO or 50 µM coumestrol for 24 h, and then washed with PBS containing protease inhibitor (Beyotime, Shanghai, China). Briefly, The equal number of cells in the two groups were incubated in a temperature range (from 50 to 71 °C) for 3 min, utilizing a thermal cycler. The cells were subsequently subjected to lysis using a lysis buffer and subsequently centrifuged at 20,000 g for 20 min at 4 °C to obtain the supernatant. The proteins within the supernatant were denatured by combining them with a loading buffer and heating in a water bath at 100 °C for a duration of 10 min. The resultant protein samples were then subjected to analysis via Western blot, utilizing an anti-DDX39A antibody (ab180857, Abcam, Shanghai, China).

### Statistical analysis

The data were expressed as the “mean ± standard deviation (SD)” of at least three independent assays. Statistical analysis was conducted through Student’s t-test and one-way analysis of variance (ANOVA) followed by Tukey’s *post-hoc* multiple comparison test. Statistical analyses were performed using GraphPad Prism (v8.0.1, GraphPad Software Inc., San Diego, CA, USA). *P* < 0.05 was considered statistically significant.

## Result

### Screening of OS-related genes

Firstly, the DEGs between OS and control samples was analyzed based on GSE39262 dataset, to preliminary screen the genes that may be involved in the tumorigenesis/progression of OS. A total of 518 DEGs were obtained, including 373 up-regulated genes and 145 down-regulated genes (Supplementary Fig. 1A-B). Subsequently, WGCNA was conducted. The soft threshold was selected as 8, and 22 modules were obtained (Supplementary Fig. 1C-D). Among them, the brown module was significantly positively correlated with OS (*r* = 0.67, *P* = 8.3e-8, Supplementary Fig. 1D), and the MM and GS of the module genes were significantly positively correlated (*r* = 0.8, *P* = 4.8e-182, Supplementary Fig. 1E). A total of 805 genes in the brown module obtained by WGCNA were cross-analyzed with the DEGs, and finally 317 genes were obtained (Supplementary Fig. 1F). GO enrichment analysis revealed that these genes were significantly enriched in 90 BP, 67 CC and 37 MF (Supplementary Table 1). BP mainly involved DNA replication initiation, mitotic spindle assembly checkpoint signaling and DNA replication; CC mainly involved CMG complex, chromosome, centromeric region and kinetochore; MF involved single-stranded DNA helicase activity, DNA replication origin binding and microtubule motor activity (*P* < 0.05, FDR < 0.05, Supplementary Fig. 2A). The results of Reactome enrichment analysis showed that these genes were involved in regulating pathways such as cell cycle, cell cycle checkpoints, and synthesis of DNA (*P* < 0.05, FDR < 0.05, Supplementary Fig. 2B). Then, KEGG enrichment analysis was conducted, and it was revealed that genes were involved in regulation of cell cycle, DNA replication, homologous recombination and p53 signaling pathway (Supplementary Fig. 2C). These results indicated that OS progression was closely related to abnormal cell proliferation and cell cycle.

### Screening of the genes related to OS prognosis

Subsequently, WGCNA was conducted based on the GSE21257 dataset, to further collect the genes involved in the metastasis of OS, and with the soft threshold set at 3, a total of 15 modules were obtained (Supplementary Fig. 3A-B). Among them, the blue module was significantly negatively correlated with metastasis (*r*=−0.52, *P* = 6.5e-5), and this module included 1413 genes (Supplementary Fig. 3B). Univariate Cox survival analysis retrieved 1,292 prognosis-related genes (*P* < 0.05, Supplementary Table 2). The cross-analysis obtained 78 genes (Supplementary Fig. 3C). GO analysis revealed that these genes were related to biological processes such as cell chemotaxis, cytokine production and cell adhesion, and were associated with cellular components such as the extracellular periphery, tertiary granular membrane and phagovesicle membrane; they were related to molecular functions such as dipeptidase activity, protein serine/threonine phosphatase activity and virus receptor activity (*P* < 0.05, Supplementary Fig. 3D). The results of signal pathway enrichment analysis showed that the genes were significantly correlated with the immune system, neutrophil degranulation and biological oxidations (Supplementary Fig. 3E). These results indicated that the metastasis of OS progression was closely related to immune microenvironment dysfunction.

### Identification of DDX39A as a potential biomarker and therapy target for OS

**Fig. 1:**
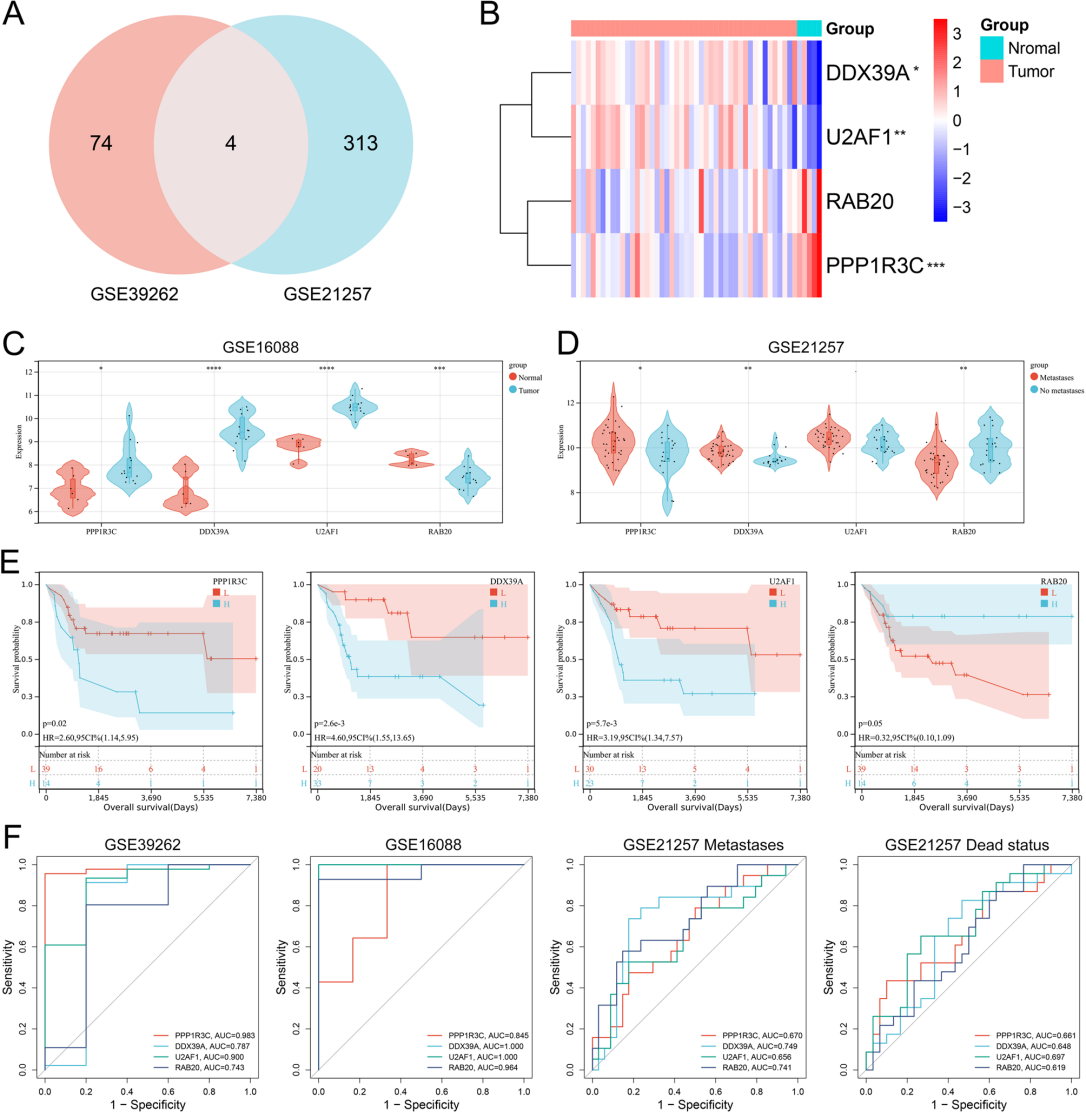
Screening of core genes in OS. **A** The Venn diagram of the common genes associated with OS occurrence (GSE39262) and OS metastasis (GSE21257). B The heat map shows the expression profile of OS core genes in GSE39262. C The violin plot shows the expression differences of the OS core gene in the control and tumor samples in GSE16088. **D** The violin plot shows the expression difference of the OS core gene in the metastatic and non-metastatic samples in GSE21257. E Kaplan-meier survival analysis of OS core genes in GSE21257. **F** ROC curves for predicting the occurrence, metastasis and survival of OS in the GSE39262, GSE16088 and GSE21257 datasets. *P < 0.05, **P < 0.01, ***P < 0.001 and ****P < 0.0001

**Fig. 2:**
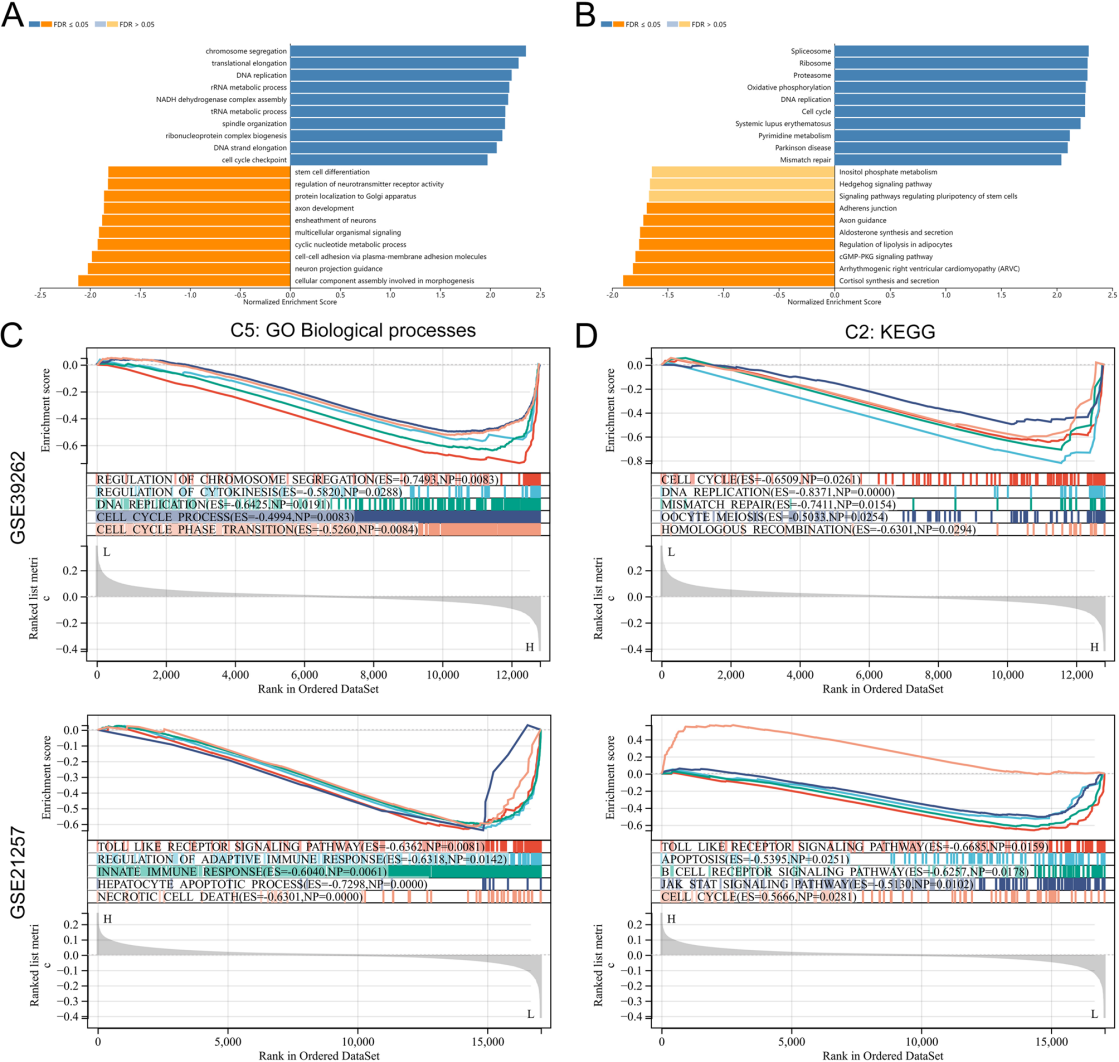
Functional analysis of DDX39A. **A**,** B** The bar chart shows the results of GO analysis and KEGG pathway enrichment analysis of DDX39A and its co-expressed genes in the TCGA-SRAC cohort. **C**,** D** The results of single-gene GSEA of DDX39A in GSE39262 and GSE21257 datasets

Cross-analysis was conducted on the genes related to OS occurrence (GSE39262) and OS metastasis (GSE21257), resulting in 4 common genes (Fig. 1A). In the GSE39262 dataset, *DDX39A* and *U2AF1* were significantly highly expressed in tumor samples, *PPP1R3* was significantly lowly expressed in tumor samples, and RAB20 was lowly expressed in tumor tissues but did not reach a significant difference (Fig. 1B). In the external dataset GSE16088, *DDX39A* and *U2AF1* were significantly highly expressed in tumor samples, and *RAB20* was significantly lowly expressed in tumor tissues, and *PPP1R3* was significantly highly expressed in tumor samples, which was inconsistent with the results in the GSE39262 dataset (Fig. 1C). In the GSE21257 dataset, *PPP1R3* and *DDX39A* were significantly highly expressed in the transferred samples, while *RAB20* was significantly lowly expressed in the transferred samples (Fig. 1D). The results of survival analysis showed that patients with high expression of *PPP1R3*, *DDX39A* and *U2AF1* had a short survival time, while patients with high expression of *RAB20* had a long survival time (Fig. 1E). The results of the ROC curve showed that these four genes had relatively good diagnostic value for the occurrence, metastasis of OS and the survival of patients, and the AUC was all greater than 0.6 (Fig. 1F). It could be concluded that only *DDX39A* was stably and highly expressed in OS (compared with the normal tissues) and metastatic tumors, and its high expression was related with poor prognosis, and it was chosen for further investigation. Next, the co-expression network of *DDX39A* was explored using the TCGA-SRAC gene expression profile data. *DDX39A* co-expressed gene were analyzed using GSEA. The results showed that *DDX39A* and its co-expressed genes were mainly involved in biological processes such as DNA replication, RNA metabolism and cell cycle (Fig. 2A-B). Subsequently, single-gene GSEA analyses of the involved biological functions and signaling pathways of *DDX39A* were conducted using the GSE39262 and GSE21257 datasets. Consistently, the results showed that *DDX39A* was associated with biological processes and signaling pathways related to the cell cycle and immune response (Fig. 2C-D).

### Immune infiltration analysis of DDX39A

**Fig. 3:**
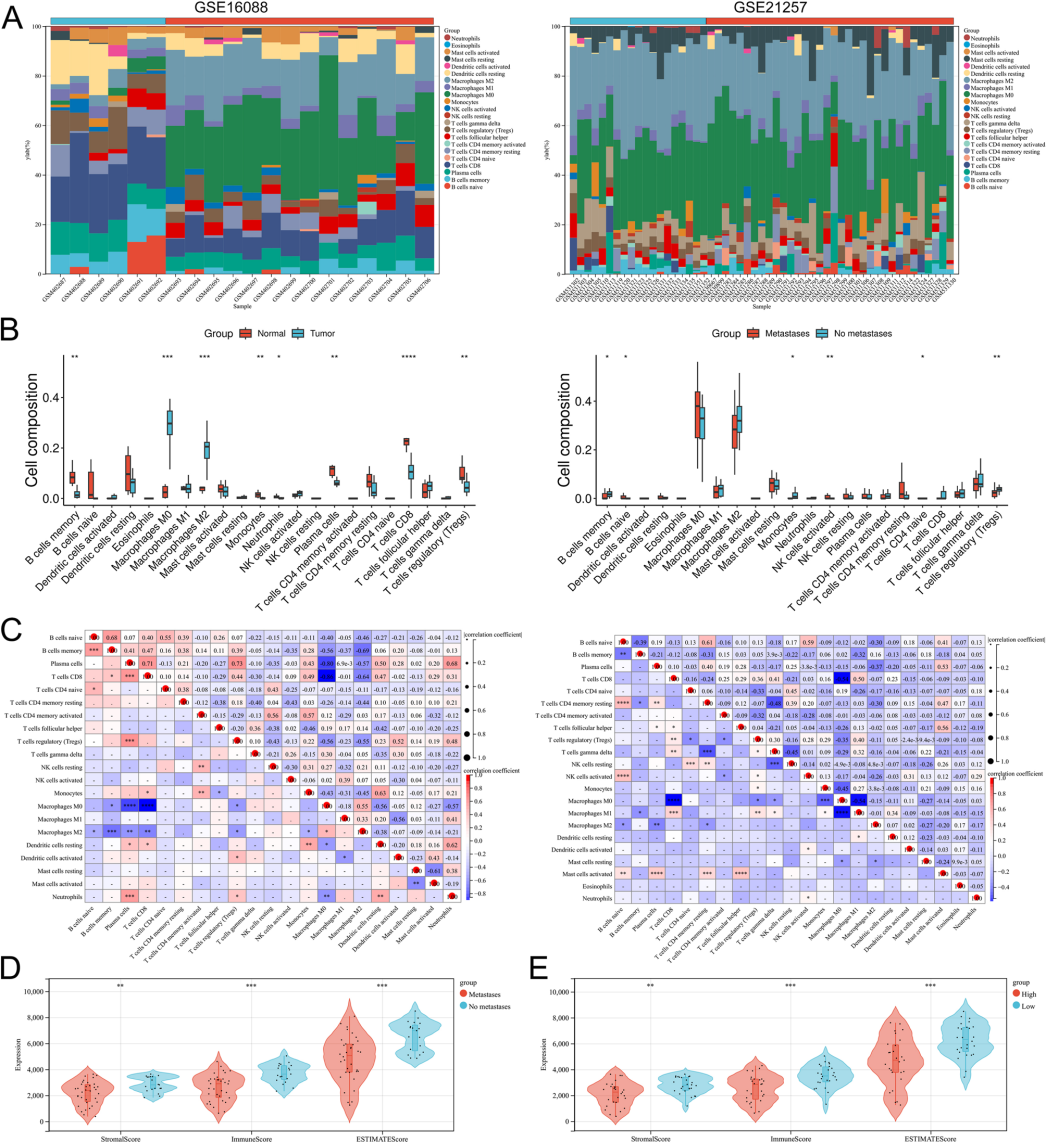
Analysis of immune infiltration. **A** The relative percentage of 22 types of immune cells in GSE16088 and GSE21257. **B** Box plots of the differences in the scores of 22 immune cells in different groups. **C** Correlation heat map of 22 types of immune cells. **D** The box plots of StromalScore, ImmuneScore and ESTIMATEscore in the metastatic and non-metastatic samples. **E** The box plots of StromalScore, ImmuneScore and ESTIMATEscore in DDX39A-high expression group and DDX39A-low expression group. *P < 0.05, **P < 0.01, ***P < 0.001 and ****P < 0.0001

The immune microenvironment has a significant impact on the diagnosis, treatment of tumors and the survival of patients. This study further analyzed the immune microenvironment of OS through the CIBERSORT algorithm. The results showed that Macrophages M0 and Macrophages M2 were significantly highly enriched in OS tumor samples. B cells memory, Monocytes, Neutrophils, Plasma cells, T cells CD8 and T cells regulatory (Tregs) were less in OS samples (Fig. 3A-B). In metastatic OS samples, B cells naive, Monocytes and NK cells activated were significantly enriched; B cells memory and T cells regulatory (Tregs) were significantly less in these samples (Fig. 3A-B). Through correlation analysis, it was found that there were multiple significant correlation pairs among immune factors (Fig. 3C). In the ESTIMATE analysis, the StromalScore, ImmuneScore and ESTIMATEscore of the metastasis group were significantly lower than those of the non-metastasis group (Fig. 3D). StromalScore, ImmuneScore and ESTIMATEscore of high *DDX39A* expression group were significantly lower than those of low DDX39A expression group (Fig. 3E). These results indicate that the occurrence and progression of OS were closely related to immune dysfunction, and the expression of *DDX39A* was associated with immunosuppression.

### DDX39A regulates the malignant phenotypes of OS cells

**Fig. 4:**
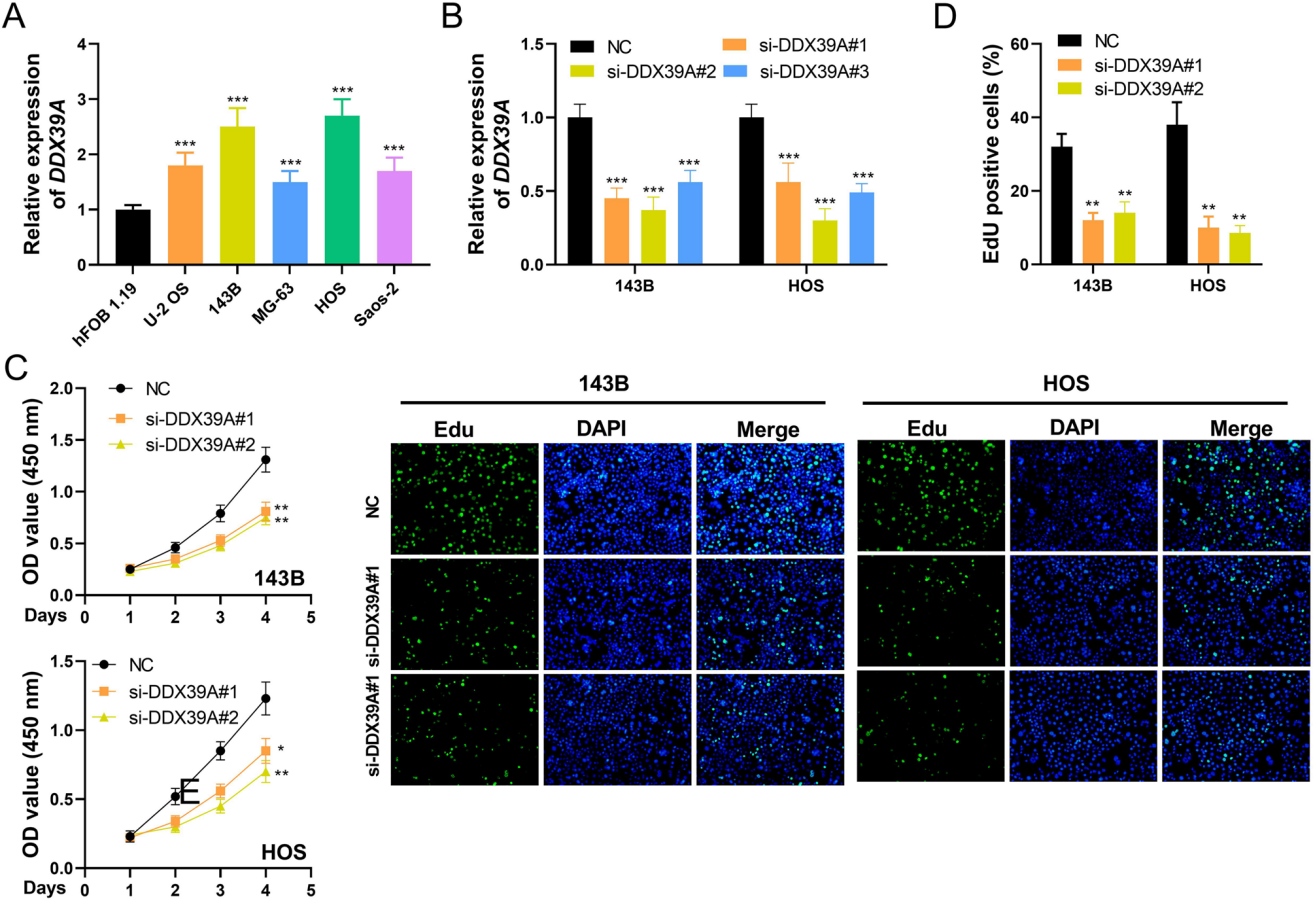
Knockdown of DDX39A inhibits the proliferation of OS cells. **A** The expression levels of *DDX39A* in OS cell lines (U-2OS, 143B, MG-63, HOS and Saos-2, etc.) and human normal osteoblast cell line hFOB 1.19 were detected by qRT-PCR. **B** The expression level of *DDX39A* in OS cells after transfection with si-DDX39A and NC was detected by qRT-PCR. **C** The CCK-8 assay was used to evaluate the viability of cells after knockdown of DDX39A. **D** The EdU experiment evaluated the proliferation ability of cells after knockdown of DDX39A. ***P < 0.001 vs. normal, hFOB 1.19 cells or NC group

**Fig. 5:**
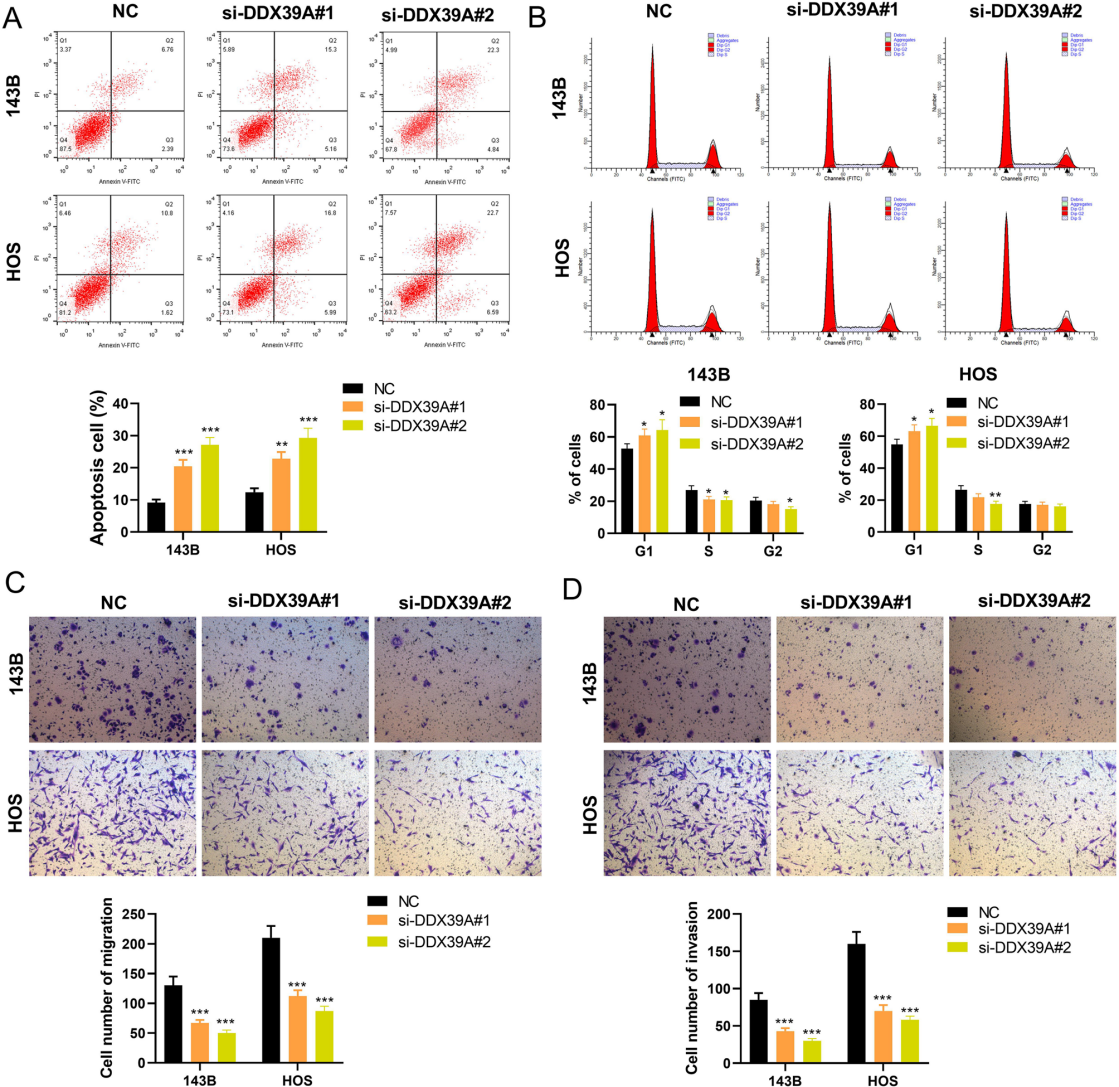
Knockdown of DDX39A promotes apoptosis and G1/S phase arrest of OS cells, and inhibits migration and invasion.** A** Flow cytometry was used to evaluate the apoptotic rates of OS cells after knockdown of DDX39A. **B** Flow cytometry was used to evaluate the cell cycle of OS cells after knockdown of DDX39A. **C**,** D** The Transwell assay was used to detect the migration and invasion abilities of OS cells after knockdown of DDX39A. *P < 0.05, **P < 0.01 and ***P < 0.001 vs. NC group

The qRT-PCR results showed that, the expression of *DDX39A* in the OS cell line was significantly higher than that in hFOB1.19 cells (Fig. 4A). To explore the effect of *DDX39A* on the phenotypes of OS cells, “loss-of-function” models were constructed in 143B and HOS cell lines, with higher expression levels of *DDX39A*. The knockdown efficiency of *DDX39A* siRNAs was detected by qRT-PCR (Fig. 4B). The CCK-8 experimental results showed that the cell viability of the *DDX39A* knockdown groups was significantly lower than that of the NC group (Fig. 4C). The EdU experimental results showed that the number of EdU positive cells in the *DDX39A* knockdown groups was significantly lower than that in the NC group (Fig. 4D). The results of flow cytometry revealed that compared with the NC group, the apoptotic level in the *DDX39A* knockdown groups increased significantly (Fig. 5A). In the *DDX39A* knockdown groups, the number of G1 cells was significantly higher than that in the NC group, and the number of cells in the S phase was significantly lower than that in the NC group (Fig. 5B). The results of the Transwell assay showed that the migration and invasion abilities of the cells in the *DDX39A* knockdown groups were lower than those of the NC group (Fig. 5C-D).

### Coumestrol inhibits the expression of DDX39A and suppress the malignant behaviors of OS cells

**Fig. 6:**
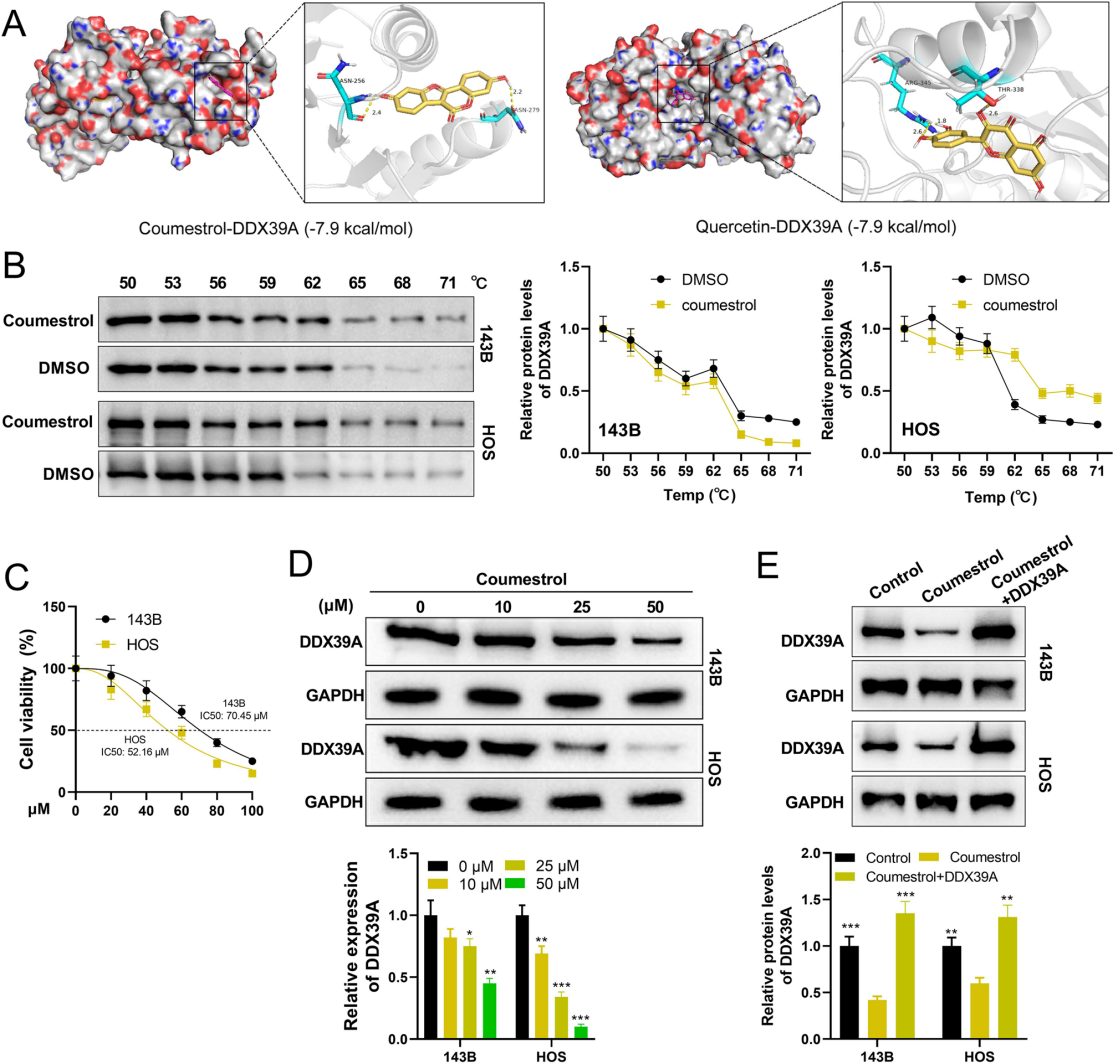
Coumestrol inhibits the expression of DDX39A.** A** Molecular docking shows the binding modes of coumestrol and quercetin with DDX39A. **B** CETSA was performed to validate the binding relationship between coumestrol and DDX39A. **C** CCK-8 was used to evaluate the toxicity of coumestrol to 143B and HOS. **D** Western blot was used to detect the effect of coumestrol on the expression of DDX39A protein in OS cells. **E** Western blot was used to detect the expression of DDX39A protein in OS cells. *P < 0.05, **P < 0.01 and ***P < 0.001 vs. 0 µM or coumestrol treatment group

**Fig. 7:**
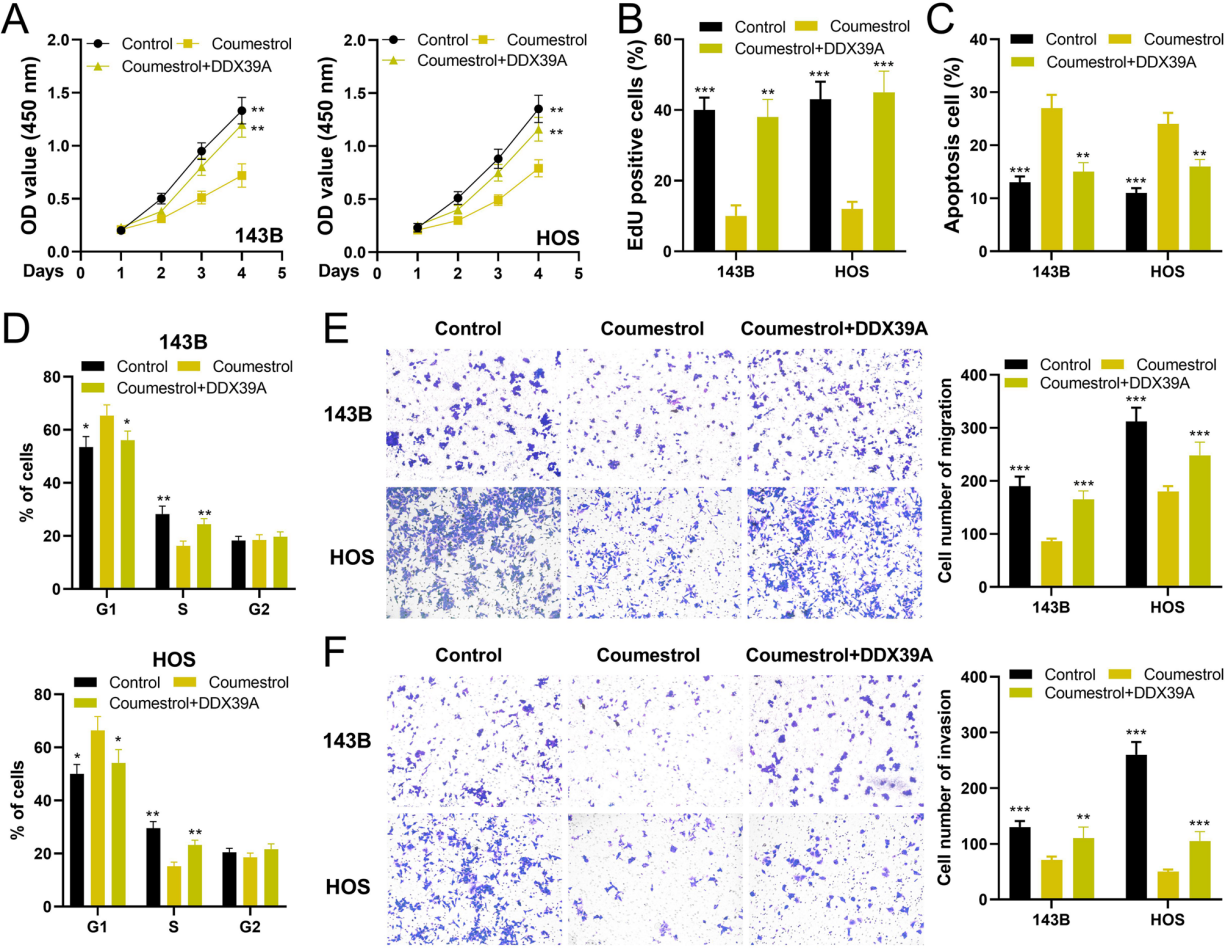
Coumestrol suppresses the malignant behaviors of OS cells. **A** CCK-8 assay evaluated the effect of coumestrol treatment and DDX39A overexpression on the viability of OS cells. **B** The EdU experiment evaluated the effect of coumestrol treatment and DDX39A overexpression on the proliferation ability of OS cells. **C** Flow cytometry was used to evaluate the effect of coumestrol treatment and DDX39A overexpression on the apoptotic level of OS cells. **D** Flow cytometry was used to evaluate the effect of coumestrol treatment and DDX39A overexpression on the cell cycle of OS cells. **E-F** The Transwell assay was used to detect the effect of coumestrol treatment and DDX39A overexpression on the migration and invasion abilities of OS cells. Data represent mean ± SD of three independent experiments. *P < 0.05, **P < 0.01 and ***P < 0.001 vs. coumestrol treatment group

The HREB database suggested that coumestrol and quercetin were the natural drugs targeting DDX39A. Molecular docking confirmed that coumestrol formed two hydrogen bonds with the ASN-256 and ASN-279 residues of DDX39A; quercetin formed three hydrogen bonds with the ARG-345 and THR-338 residues of DDX39A, both of which had good binding activity (Fig. 6A). The CETSA experimental results indicate that DDX39A can bind to coumestrol (Fig. 6B). 143B and HOS cells were treated with different concentrations of coumestrol for 24 h. The cell viability gradually decreased with the increase of drug concentration. The IC_50_ values of 143B and HOS cells were 70.45 µM and 52.16 µM, respectively (Fig. 6C). Subsequently, the effect of coumestrol on the DDX39A protein was detected by western blot. The results showed that with the increase of coumestrol concentration, the protein expression level of DDX39A gradually decreased; moreover, when the concentration is 50 µM, coumestrol has a better inhibitory effect on DDX39A (Fig. 6D). These data imply that coumestrol has two inhibitory effects on DDX39A (both on expression and activity). To detect whether coumestrol affected the phenotypes of OS cells by regulating the expression of DDX39A, the *DDX39A* overexpression cell line was constructed, and then OS cells were treated with 50 µM coumestrol (Fig. 6E). It was found that the viability of cells in coumestrol treatment group was significantly lower than that in the control group; compared with the coumestrol treatment group, the cell viability in coumestrol + *DDX39A* group was significantly increased (Fig. 7A). The number of EdU positive cells in coumestrol treatment group was significantly lower than that in the control group, and the number of EdU positive cells in the coumestrol + *DDX39A* group was significantly higher than that in the coumestrol treatment group (Fig. 7B). Compared with the control group, coumestrol significantly promoted the apoptosis of OS cells, which was reversed by DDX39A restoration (Fig. 7C). Also, coumestrol treatment significantly increased the proportion of cells in the G1 phase and reduced the proportion of cells in the S phase, while overexpression of *DDX39A* significantly reversed this phenomenon (Fig. 7D). Transwell assay showed that coumestrol significantly inhibited the migration and invasion of OS cells, and the overexpression of *DDX39A* partially reversed the effect of coumestrol on OS cells (Fig. 7E-F). When the cells were cultured in serum-free medium, the inhibitory rate of viability of OS cells is less than 20%, which was also validated with flow cytometry (Supplementary Fig. 4A&B). Considering the inhibitory rate of motility was much high than that of the viability, we believed that the reduced motility was not secondary to reduced viability. Additionally, the protein-protein interaction (PPI) network of DDX39A was plotted with the GeneMANIA database and the STRING database (Supplementary Fig. 5A), and KEGG enrichment analysis was performed based on the proteins interacting with DDX39A, and the results showed these proteins were probably associated with transcription and RNA processing (Supplementary Fig. 5B&C). Interestingly, qRT-PCR showed that, coumestrol markedly repressed the expression of some proteins interacting with DDX39A (including *SARNP*, *DDX39B*, *ALYREF*, *THOC1*, *THOC2*), which was reversed by the restoration of DDX39A (Supplementary Fig. 5D). These data further implied that coumestrol repressed the malignant biological behaviors of OS cells via modulating DDX39A-related pathway.

## Discussion

In this study, the genes related to OS were identified respectively by DEGs analysis, univariate Cox analysis and WGCNA. These genes were mainly related with DNA replication, cell cycle and p53 signaling pathway. p53 is a well-known tumor suppressor, and it can directly regulate the expression of over 500 genes, many of which are involved in modulating biological processes such as cell cycle arrest, cell senescence, apoptosis, cell death and DNA damage repair [16]. Mutations in the TP53 gene are the most common single-gene alterations in human cancers, driving tumorigenesis and disease progression, and also important targets for cancer treatment [17]. p53 deficiency and cell cycle progression activation have been reported to contribute to OS progression [18]. Our study further emphasized the importance of p53 pathway in OS, which is consistent with the previous reports [16–18]. Tumor microenvironment contributes to cancer progression, and immune cell infiltration is closely related to the spread and metastasis of OS [19]. B cells and T cells are crucial for killing tumor cells in lymph nodes, and tumor cells can reciprocally affect cytotoxic T cells, thereby leading to immune escape [20]. The present work demonstrated that the enrichment of B cells memory and T cells regulatory (Tregs) in OS tumor samples and metastatic samples were significantly higher than those in non-metastatic samples, which is consistent with the previous reports [21, 22]. Monocytes can produce tumor-killing mediators and stimulate natural killer (NK) cells. In this study, the recruitment of monocytes in metastatic OS samples was significantly lower than that in non-metastatic samples, and consistently, a previous study has reported that a higher proportion of monocytes is associated with a better prognosis in patients with OS [23]. In OS samples, the enrichment of macrophages M0 and M2 were higher, which is also consistent with the results of some previous studies [24, 25].

Further analysis revealed four genes related to OS prognosis, including *DDX39A*, *U2AF1*, *PPP1R3* and *RAB20*. *PPP1R3* gene is located on chromosome 7q31 and encodes protein phosphatase 1 (regulatory 3), participating in the regulation of cell division and growth. It is reported that PPP1R3 is associated with lymph node and liver metastases of colorectal cancer [26]. U2 small nuclear RNA auxiliary factor 1 (U2AF1) regulates the RNA splicing during gene expression, and its mutations are often observed in myelodysplastic syndrome, primary myelofibrosis and other solid tumors [27]. In the results of this study, we found that U2AF1 was significantly highly expressed in OS samples, and the survival time of patients with its high expression was shorter. This might be that U2AF1 promotes cancer progression through its non-classical role in translation regulation [28]. Ras-related binding proteins (RABs) are dysregulated in various cancers and play a key role in regulating the cell cycle process and immune cell infiltration [29]. RAB20 has been reported to be upregulated in colorectal cancer and pancreatic cancer [30], and the overexpression of RAB20 is associated with liver metastasis of colorectal cancer [31]. In addition, RAB20 participates in cell cycle regulation [29]. However, in hepatocellular carcinoma (HCC), RAB20 is lowly expressed. Its dysregulation can promote the occurrence of HCC, and the tumor suppressor effect of RAB20 may be attributed by the release of extracellular vesicles [31]. In this study, it was revealed that RAB20 was significantly lowly expressed in OS samples and metastatic samples, and patients with high expression of RAB20 had a longer survival period, indicating that RAB20 may exert tumor-suppressive properties in OS.

DDX39A, as a member of the DEAD-box RNA helicase family of nucleic acid-binding proteins, is involved in regulating cytoplasmic mRNA localization, genomic integrity, as well as mitosis and cytoplasmic division processes [32]. DDX39A promotes the growth and metastasis of hepatocellular carcinoma by facilitating the accumulation of β-catenin in the nucleus and participating in the activation of Wnt/β-catenin pathway [33, 34]. DDX39A, as an RNA splicing factor, may increase RNA helicase activity, promote the splicing and transport of mRNA of epithelial-mesenchymal transition (EMT)-related genes, and increase their expression, and promotes cancer metastasis [35, 36]. DDX39A promotes the proliferation and invasion of tongue squamous cell carcinoma cells [37]. In breast cancer, the increased expression of DDX39 mRNA is associated with the poor prognosis of ER-positive breast cancer, and inhibiting DDX39 sensitize MCF-7 cells to doxorubicin [38]. Additionally, DDX39A facilitates the proliferation, 3D organoid formation and cell cycle progression of colorectal cancer cells [39]. The role of DDX39A is rarely reported by the previous studies. The present work demonstrated that DDX39A was highly expressed in OS, and the high expression of *DDX39A* was significantly associated with a shorter survival time of OS patients. Therefore, DDX39A may be used as a potential prognostic biomarker for OS. Also, we reported that *DDX39A* knockdown could inhibit multiple malignant phenotypes of OS cells, including proliferation, migration, invasion, cell cycle progression and anti-apoptosis. To our best knowledge, this work is the first study reporting the oncogenic properties of DDX39A in OS.

Coumestrol is a kind of phytoestrogen. These compounds have been reported to exhibit both pro-apoptotic and anti-proliferative properties on cancer cells [40]. The tumor-suppressive properties of coumestrol has been reported in multiple human malignancies such as colorectal cancer, breast cancer and skin carcinoma [41–43]. However, its pharmacological effects on OS cells are rarely investigated. In the present work, it was confirmed through the HERB database and molecular docking that DDX39A and coumestrol had good binding ability. Furthermore, in vitro assays indicated that coumestrol could significantly inhibit the malignant behaviors of OS cells, while the overexpression of DDX39A partially reversed this phenomenon. These results indicate that coumestrol exerts an anti-OS effect by regulating the expression of DDX39A. However, this study still has certain limitations. For example, in vivo experiments are still needed to validate the biological functions of coumestrol and DDX39A.

## Conclusion

DDX39A is a prognostic biomarker for OS and is highly expressed in OS. DDX39A facilitates the malignant biological behaviors of OS cells. Coumestrol warrants further preclinical evaluation as a potential DDX39A inhibitor in OS.

## Supplementary Information

Supplementary Material 1. Supplementary Table 1. The results of GO analysis of OS-related genes.

Supplementary Material 2. Supplementary Table 2. The prognosis-realted genes in OS identified by univariate Cox survival analysis.

Supplementary Material 3. Supplementary Fig. 1. Screening of OS-related genes. (A) The volcano map shows the DEGs in the GSE39262 dataset. The blue dots represent down-regulated expression genes, the red dots represent up-regulated expression genes, and the grey dots represent insignificantly expressed genes. (B) The heat map shows the expression profiles of the top 10 down-regulated and up-regulated genes in the samples from the GSE39262 dataset. Blue represents the control sample and red represents the tumor sample. (C) Scale independence and average connectivity in WGCNA. (D) The heat map shows the correlation between gene modules and phenotypes in WGCNA. (E) Scatter plot of GS and MM relationship of genes in the brown module. (F) The Venn diagram of the common genes of DEGs and crucial genes identified by WGCNA.

Supplementary Material 4. Supplementary Fig. 2. Functional enrichment analysis of OS-related genes. A The bar chart shows the results of GO analysis, including the top 15 items in the three aspects of BP, CC, and MF (sorted by the number of genes). B The bar chart shows the results of Reactome pathway enrichment analysis. C The bar chart shows the results of KEGG pathway enrichment analysis.

Supplementary Material 5. Supplementary Fig. 3. Screening of genes related to OS prognosis. (A) Scale independence and average connectivity in WGCNA. (B) The heat map shows the correlation between gene modules and phenotypes. (C) The Venn diagram shows the common genes of the genes identified by univariate Cox analysis and the genes identified by WGCNA. (D) The bar chart shows the results of GO analysis, including the top 15 items in the three aspects of BP, CC, and MF (sorted by the number of genes). E The bar chart shows the results of Reactome and KEGG pathway enrichment analysis.

Supplementary Material 6. Supplementary Fig. 4. Coumestrol represses the viability of OS cells cultured in serum-free medium. (A) CCK-8 was used to evaluate the toxicity of coumestrol to 143B and HOS in serum-free medium. (B) Flow cytometry was used to evaluate the effect of 50 µM coumestrol treatment on the apoptotic level of OS cells. Data represent mean ± SD of three independent experiments. **P < 0.01.

Supplementary Material 7. Supplementary Fig. 5 Analysis of downstream interacting proteins of DDX39A. (A) PPI network of DDX39A interacting proteins in GeneMANIA database. (B) PPI network of DDX39A interacting proteins in STRING database. (C) Bubble chart displaying KEGG enrichment analysis results of DDX39A interacting protein. (D) qRT-PCR was used to detect the effect of coumestrol on the expression of DDX39A interacting proteins (SARNP, DDX39B, ALYREF, THOC1 and THOC2) in OS cells. Data represent mean ± SD of three independent experiments. ***P < 0.01.

Supplementary Material 8.

## Data Availability

The data used to support the findings of this study are available from the corresponding author upon request.
