# Supplementary figures and images for: DExD-box helicase 39 A, targeted by coumestrol, facilitates the malignant behaviors of osteosarcoma cells
Source: Hereditas. 2025 Oct 28;162:218. doi: 10.1186/s41065-025-00588-0 (PMC12570790; doi:10.1186/s41065-025-00588-0)

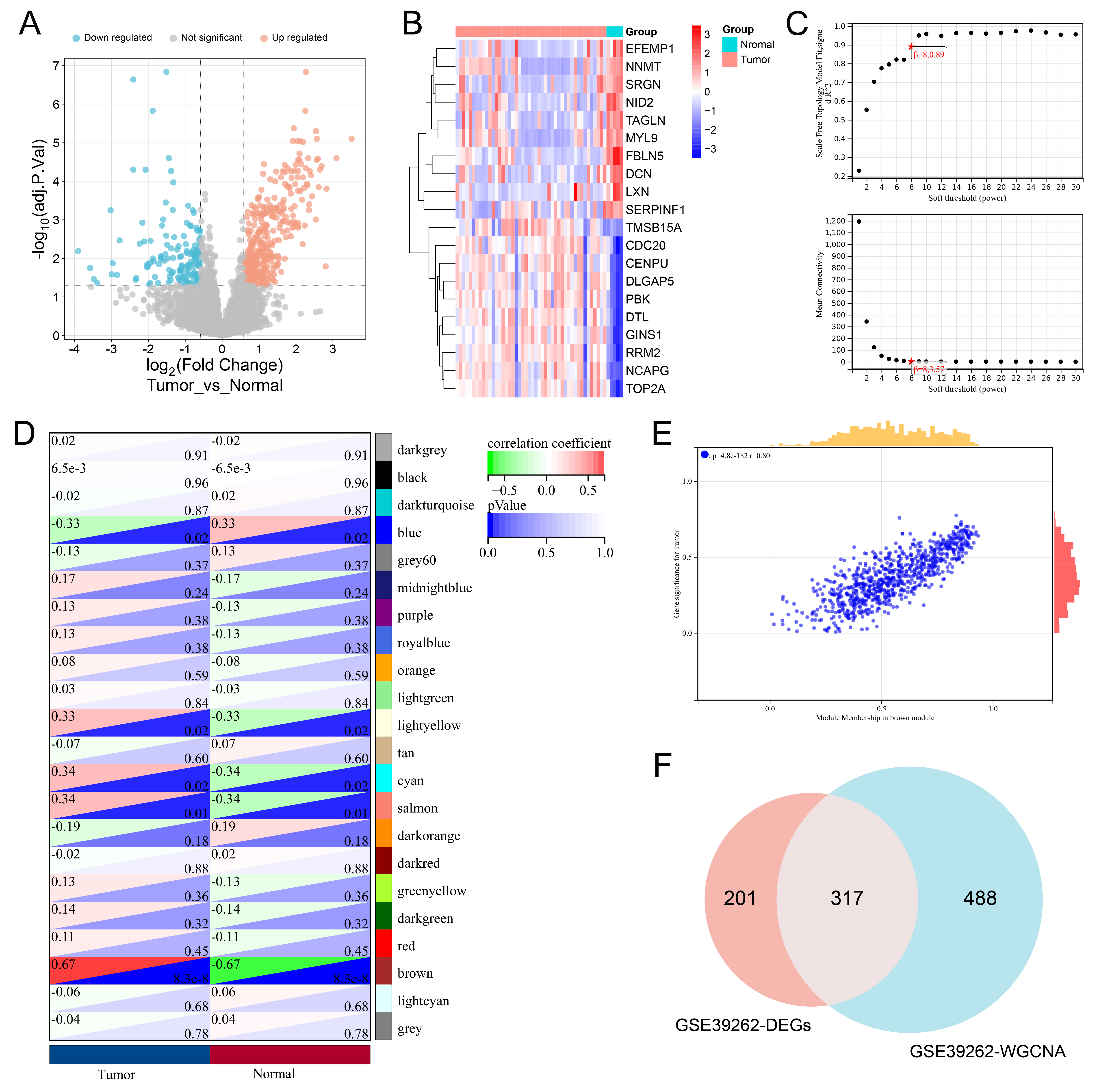

Supplement: Supplementary file 3 — Supplementary Material 3. Supplementary Fig. 1. Screening of OS-related genes. (A) The volcano map shows the DEGs in the GSE39262 dataset. The blue dots represent down-regulated expression genes, the red dots represent up-regulated expression genes, and the grey dots represent insignificantly expressed genes. (B) The heat map shows the expression profiles of the top 10 down-regulated and up-regulated genes in the samples from the GSE39262 dataset. Blue represents the control sample and red represents the tumor sample. (C) Scale independence and average connectivity in WGCNA. (D) The heat map shows the correlation between gene modules and phenotypes in WGCNA. (E) Scatter plot of GS and MM relationship of genes in the brown module. (F) The Venn diagram of the common genes of DEGs and crucial genes identified by WGCNA. [file 41065_2025_588_MOESM3_ESM.jpg]

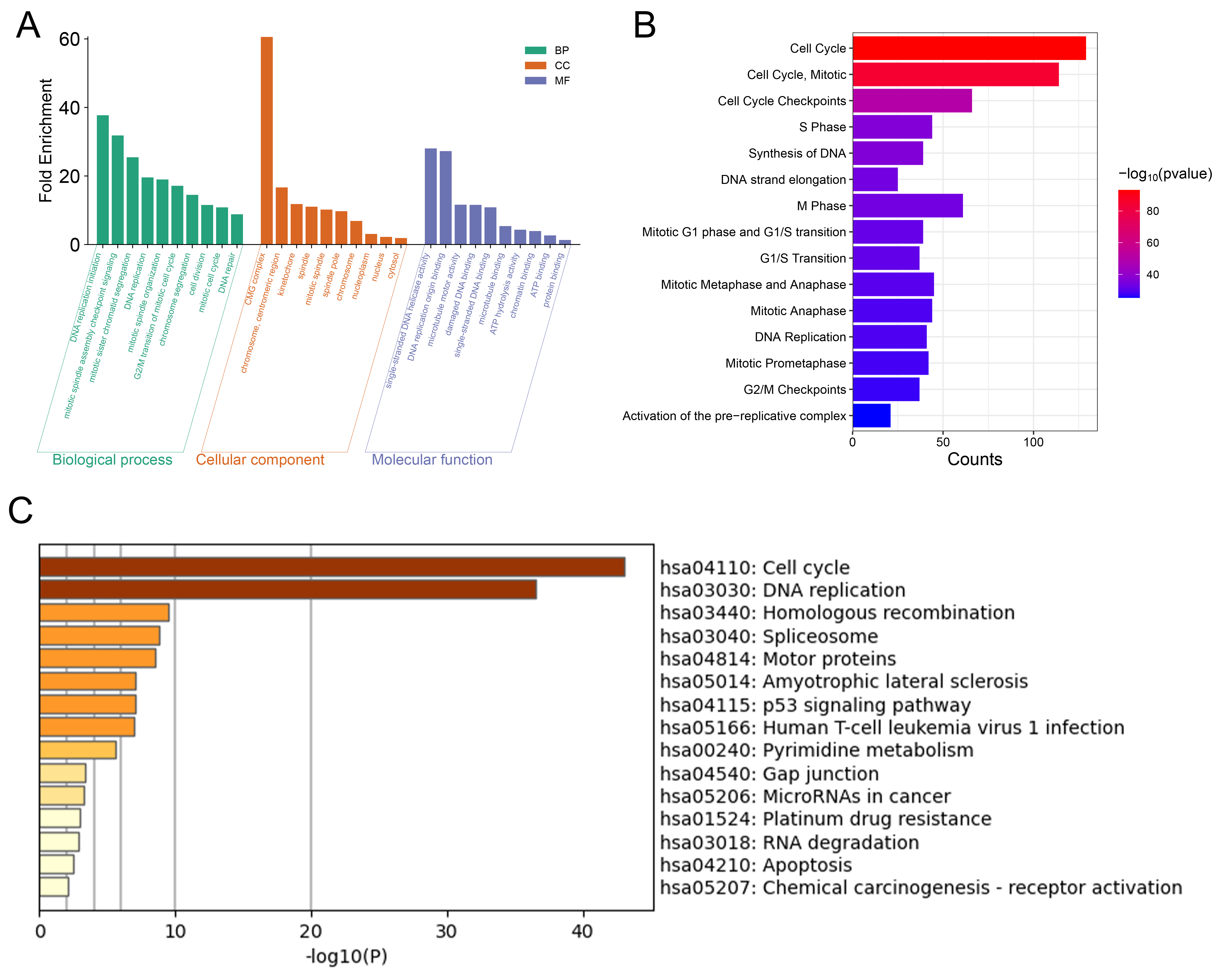

Supplement: Supplementary file 4 — Supplementary Material 4. Supplementary Fig. 2. Functional enrichment analysis of OS-related genes. A The bar chart shows the results of GO analysis, including the top 15 items in the three aspects of BP, CC, and MF (sorted by the number of genes). B The bar chart shows the results of Reactome pathway enrichment analysis. C The bar chart shows the results of KEGG pathway enrichment analysis. [file 41065_2025_588_MOESM4_ESM.jpg]

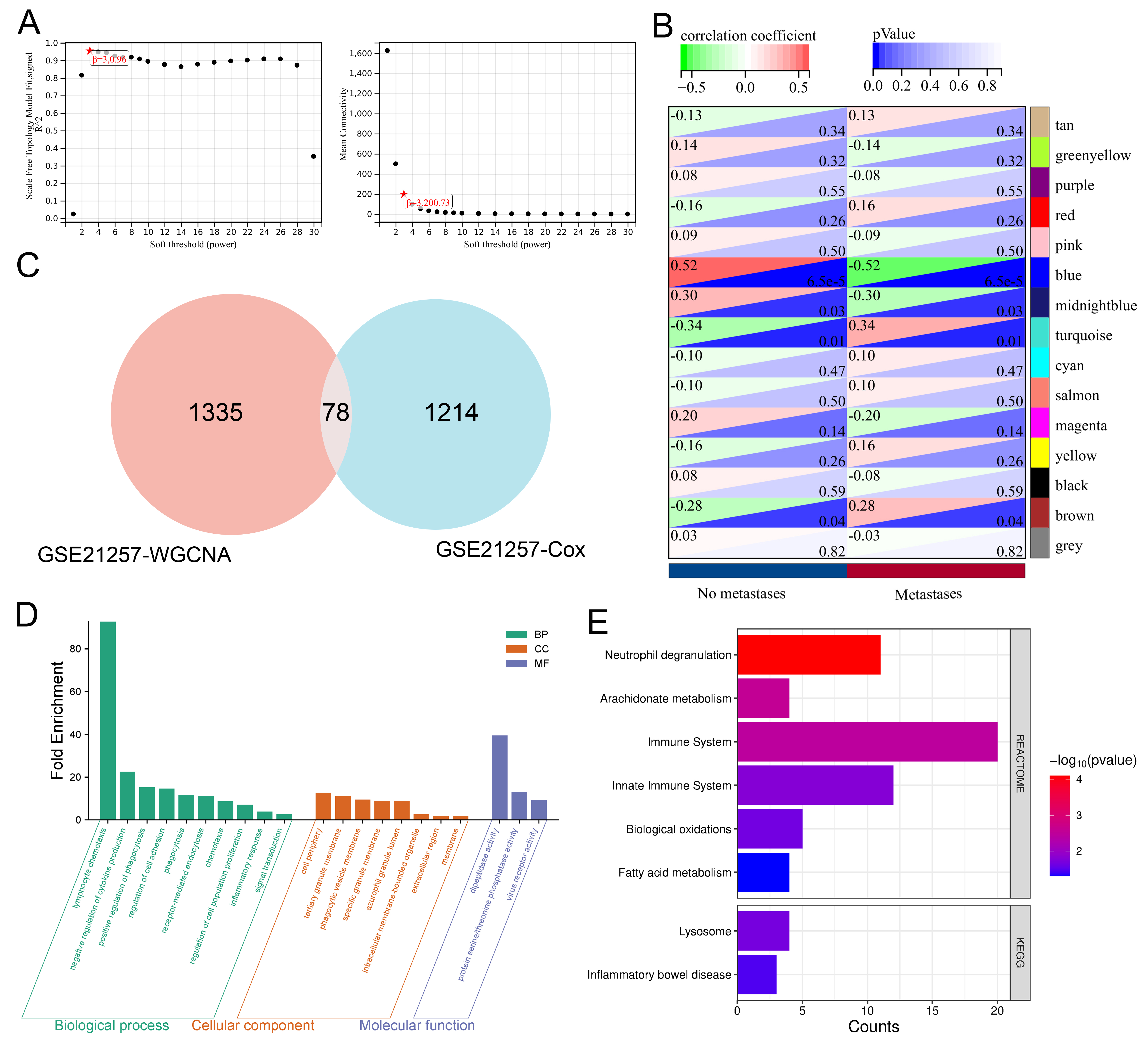

Supplement: Supplementary file 5 — Supplementary Material 5. Supplementary Fig. 3. Screening of genes related to OS prognosis. (A) Scale independence and average connectivity in WGCNA. (B) The heat map shows the correlation between gene modules and phenotypes. (C) The Venn diagram shows the common genes of the genes identified by univariate Cox analysis and the genes identified by WGCNA. (D) The bar chart shows the results of GO analysis, including the top 15 items in the three aspects of BP, CC, and MF (sorted by the number of genes). E The bar chart shows the results of Reactome and KEGG pathway enrichment analysis. [file 41065_2025_588_MOESM5_ESM.jpg]

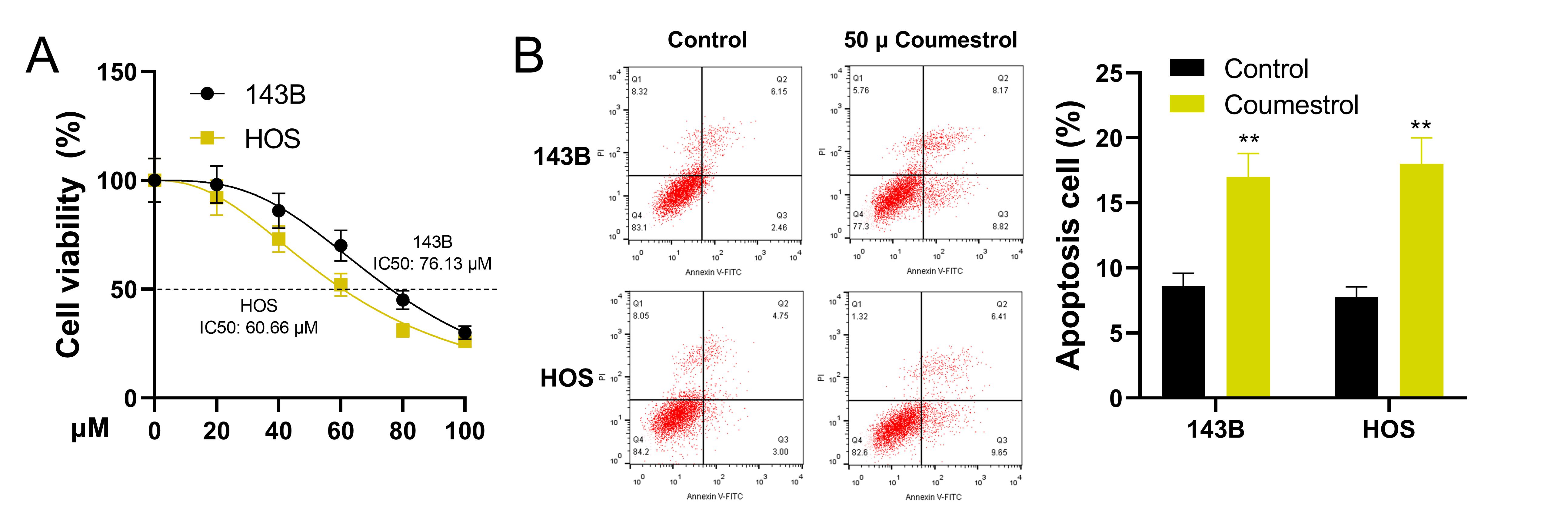

Supplement: Supplementary file 6 — Supplementary Material 6. Supplementary Fig. 4. Coumestrol represses the viability of OS cells cultured in serum-free medium. (A) CCK-8 was used to evaluate the toxicity of coumestrol to 143B and HOS in serum-free medium. (B) Flow cytometry was used to evaluate the effect of 50 µM coumestrol treatment on the apoptotic level of OS cells. Data represent mean ± SD of three independent experiments. **P < 0.01. [file 41065_2025_588_MOESM6_ESM.jpg]

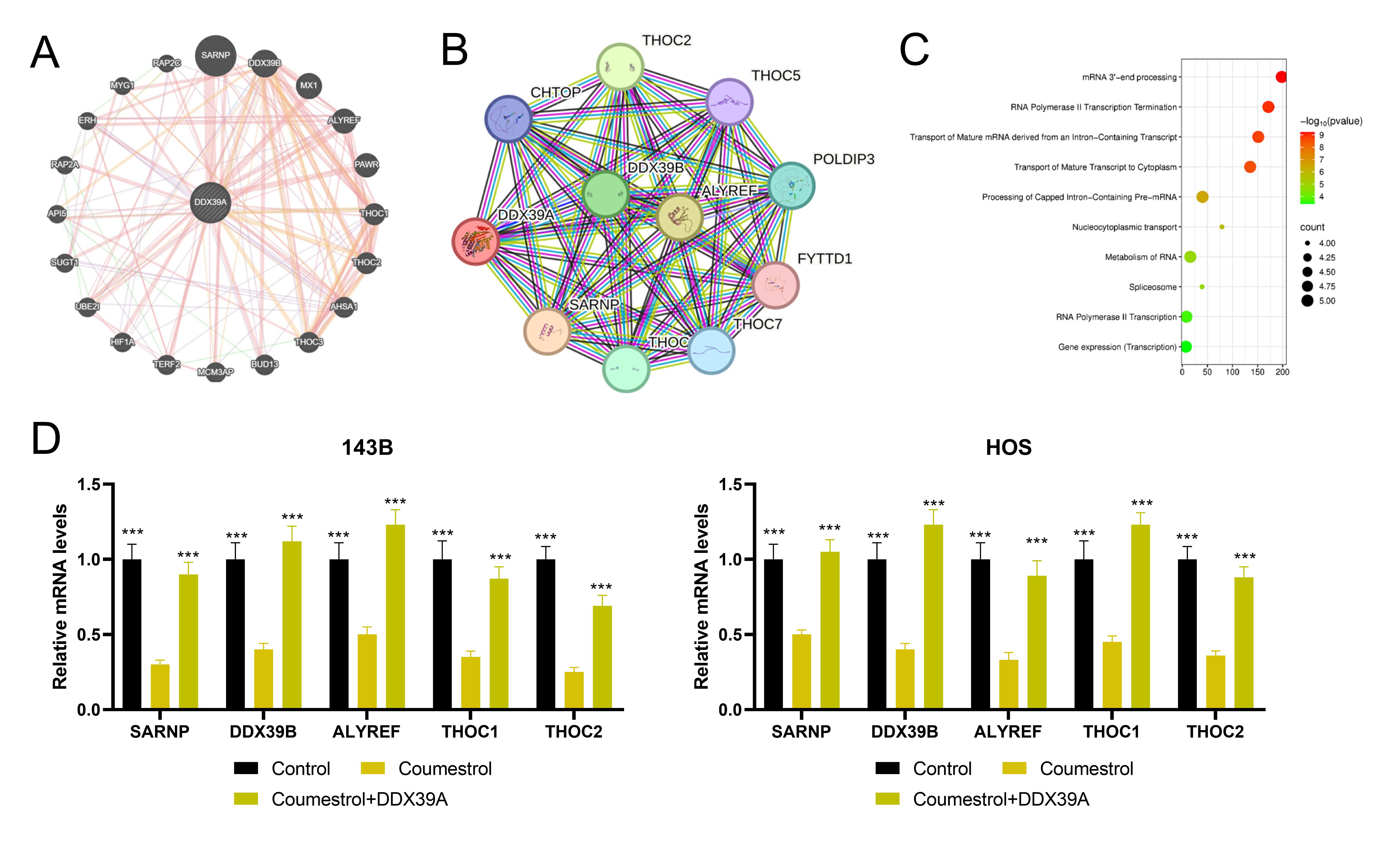

Supplement: Supplementary file 7 — Supplementary Material 7. Supplementary Fig. 5 Analysis of downstream interacting proteins of DDX39A. (A) PPI network of DDX39A interacting proteins in GeneMANIA database. (B) PPI network of DDX39A interacting proteins in STRING database. (C) Bubble chart displaying KEGG enrichment analysis results of DDX39A interacting protein. (D) qRT-PCR was used to detect the effect of coumestrol on the expression of DDX39A interacting proteins (SARNP, DDX39B, ALYREF, THOC1 and THOC2) in OS cells. Data represent mean ± SD of three independent experiments. ***P < 0.01. [file 41065_2025_588_MOESM7_ESM.jpg]
