# Supplementary material for: DExD-box helicase 39 A, targeted by coumestrol, facilitates the malignant behaviors of osteosarcoma cells
Source: Hereditas. 2025 Oct 28;162:218. doi: 10.1186/s41065-025-00588-0 (PMC12570790; doi:10.1186/s41065-025-00588-0)

Figure 6B

143B

Coumestrol


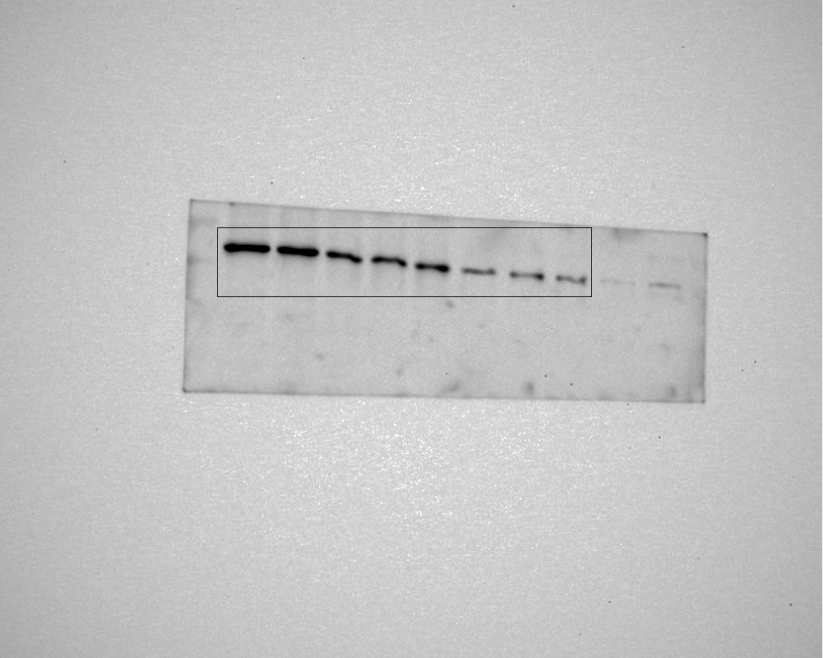


DMSO


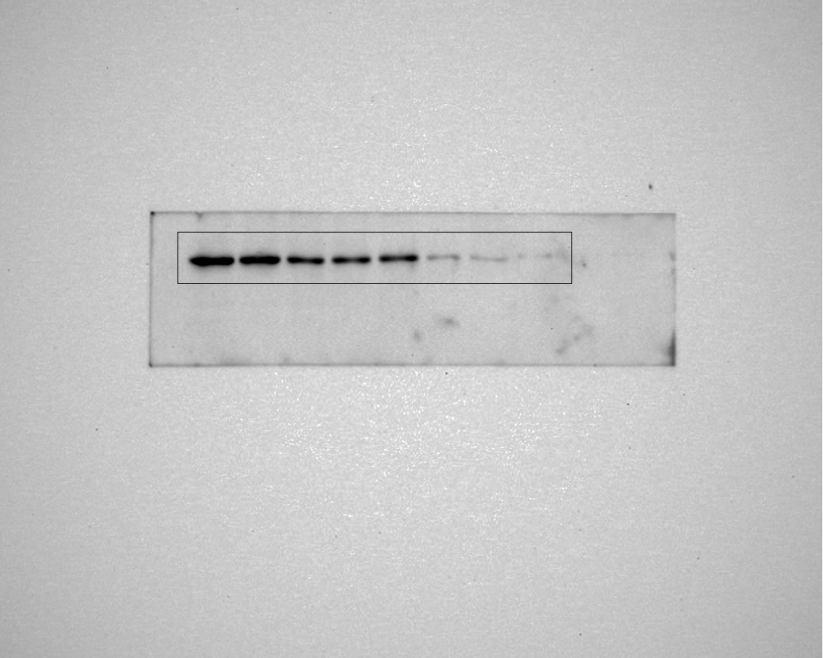


HOS

Coumestrol


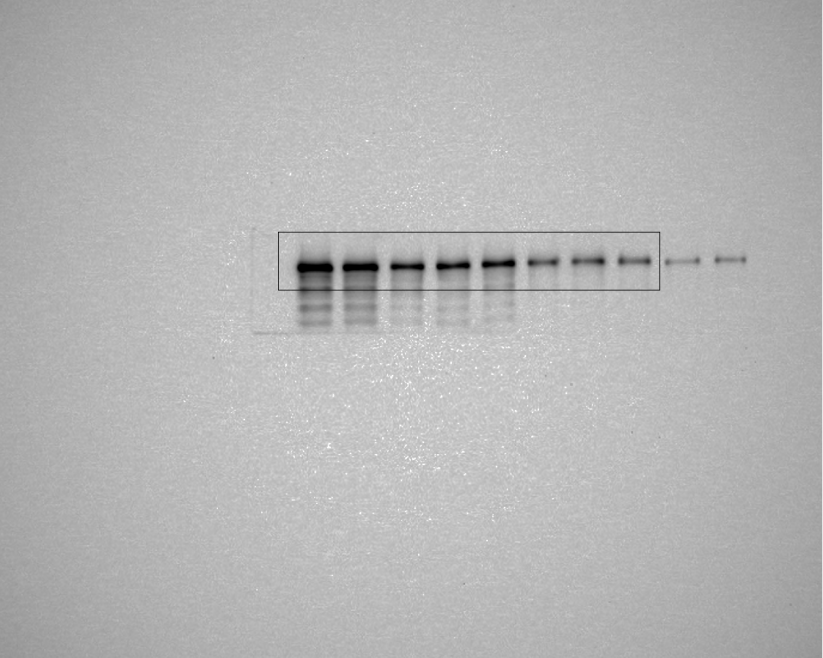


DMSO


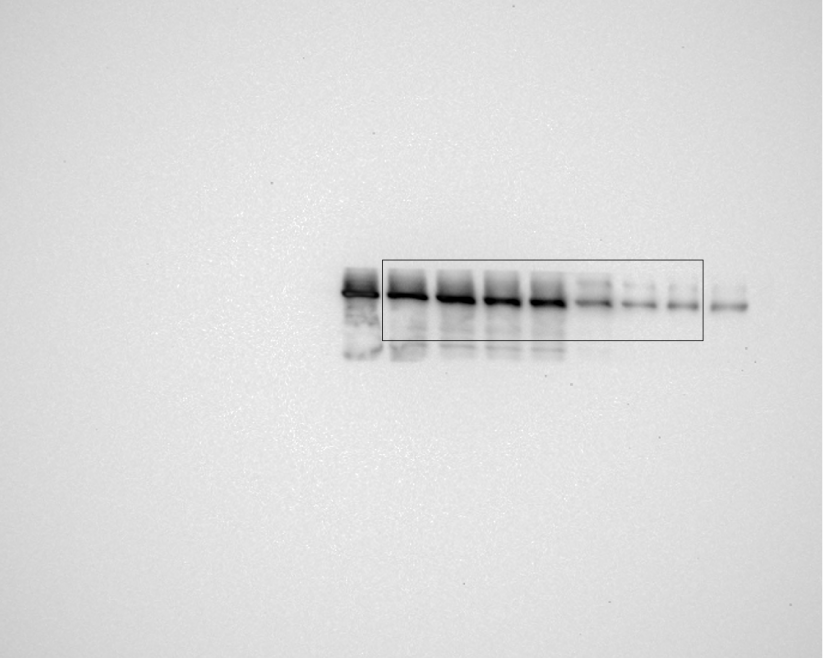


Figure 6D

143B

DDX39A


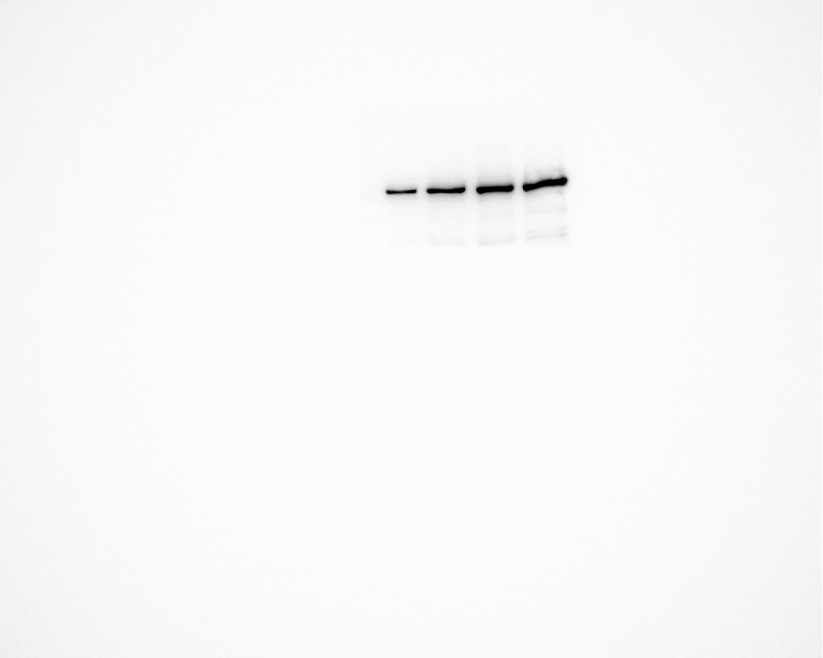


GAPDH


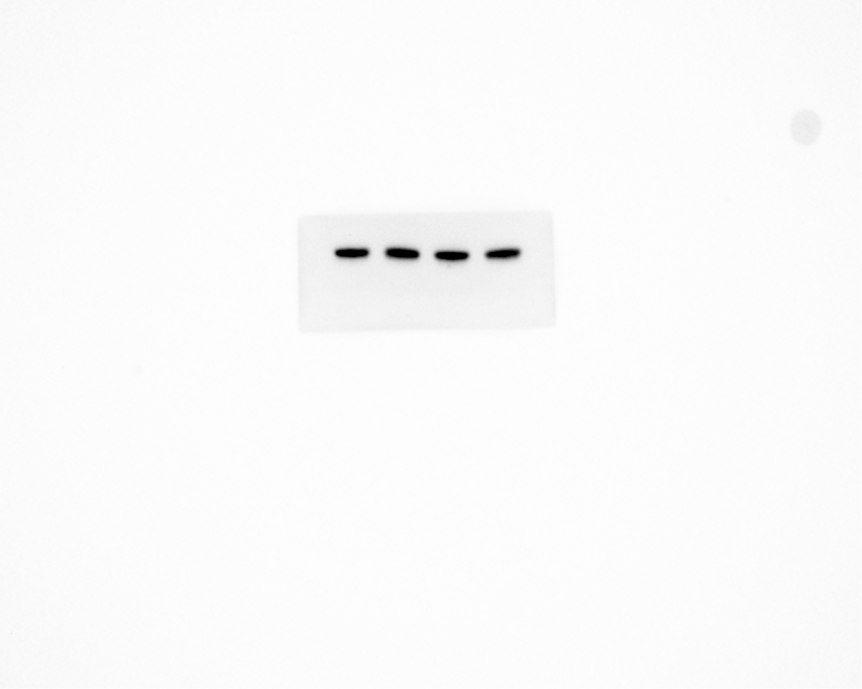


HOS

DDX39A


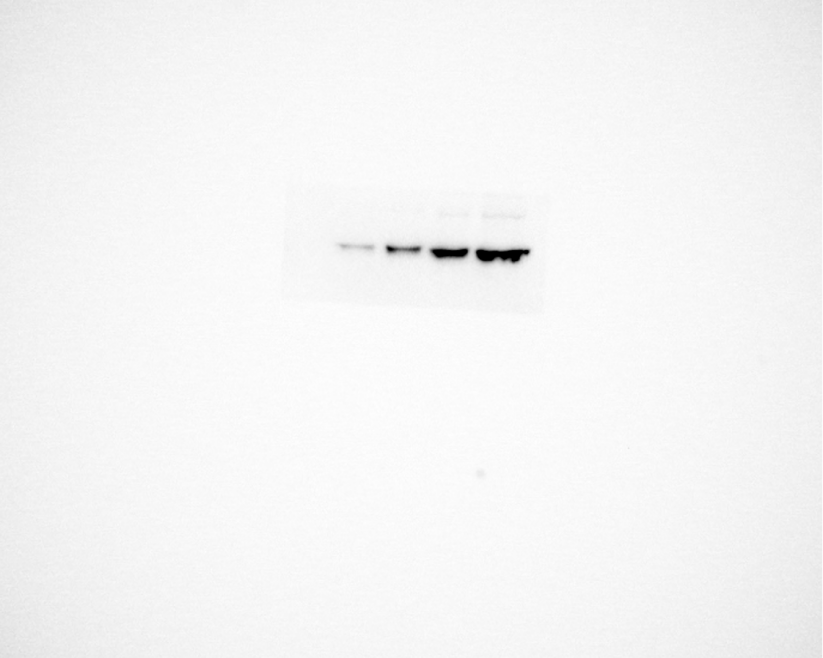


GAPDH


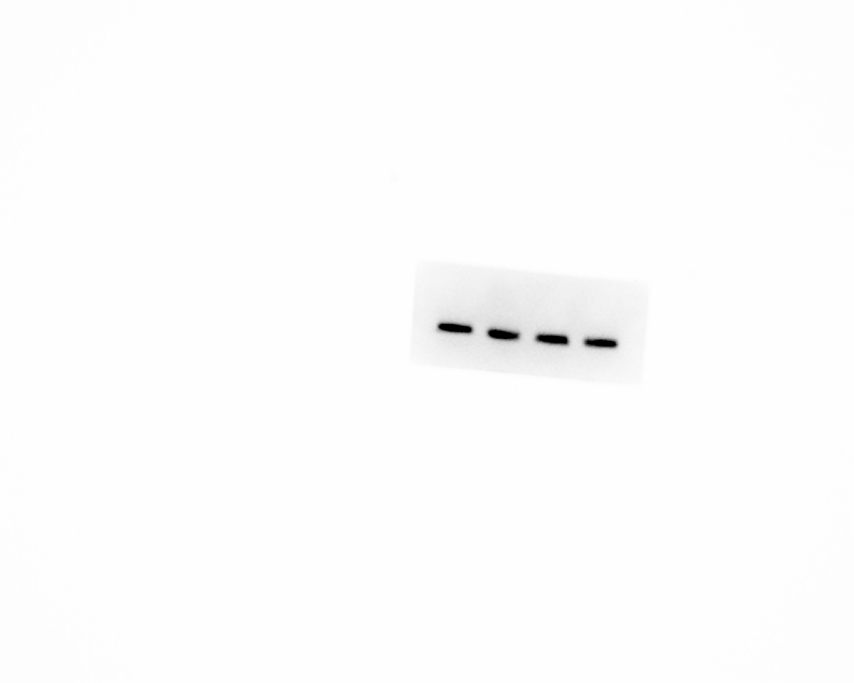


Figure 6E

143B

DDX39A


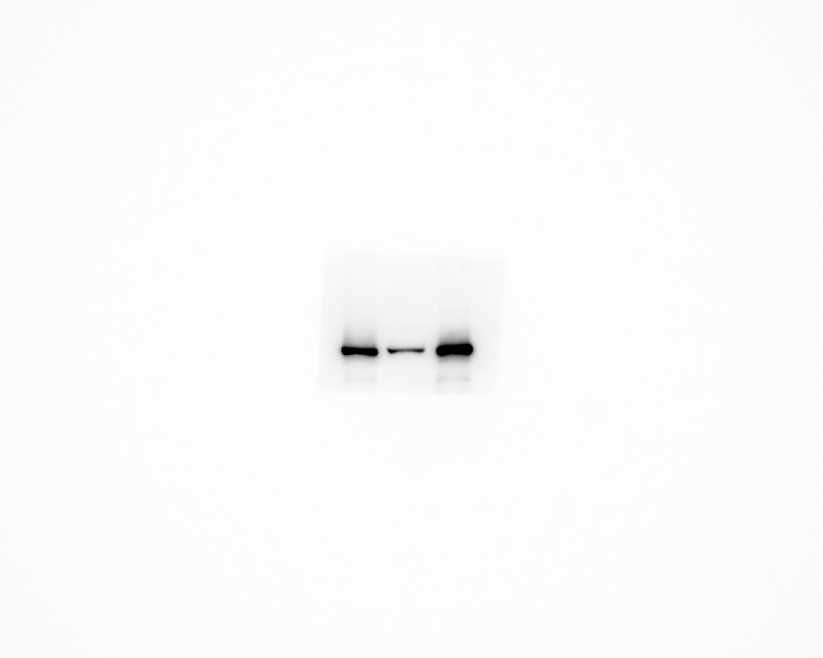


GAPDH


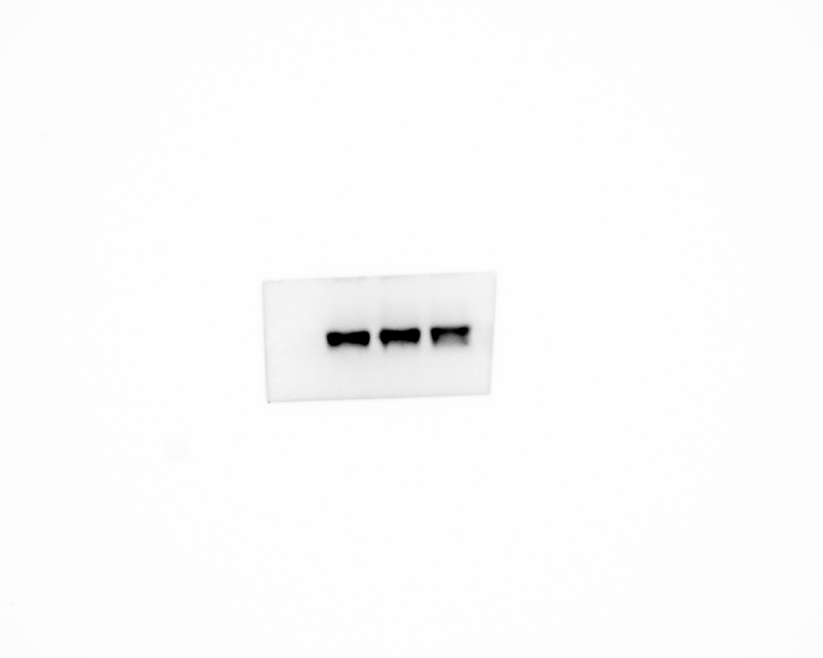


HOS

DDX39A


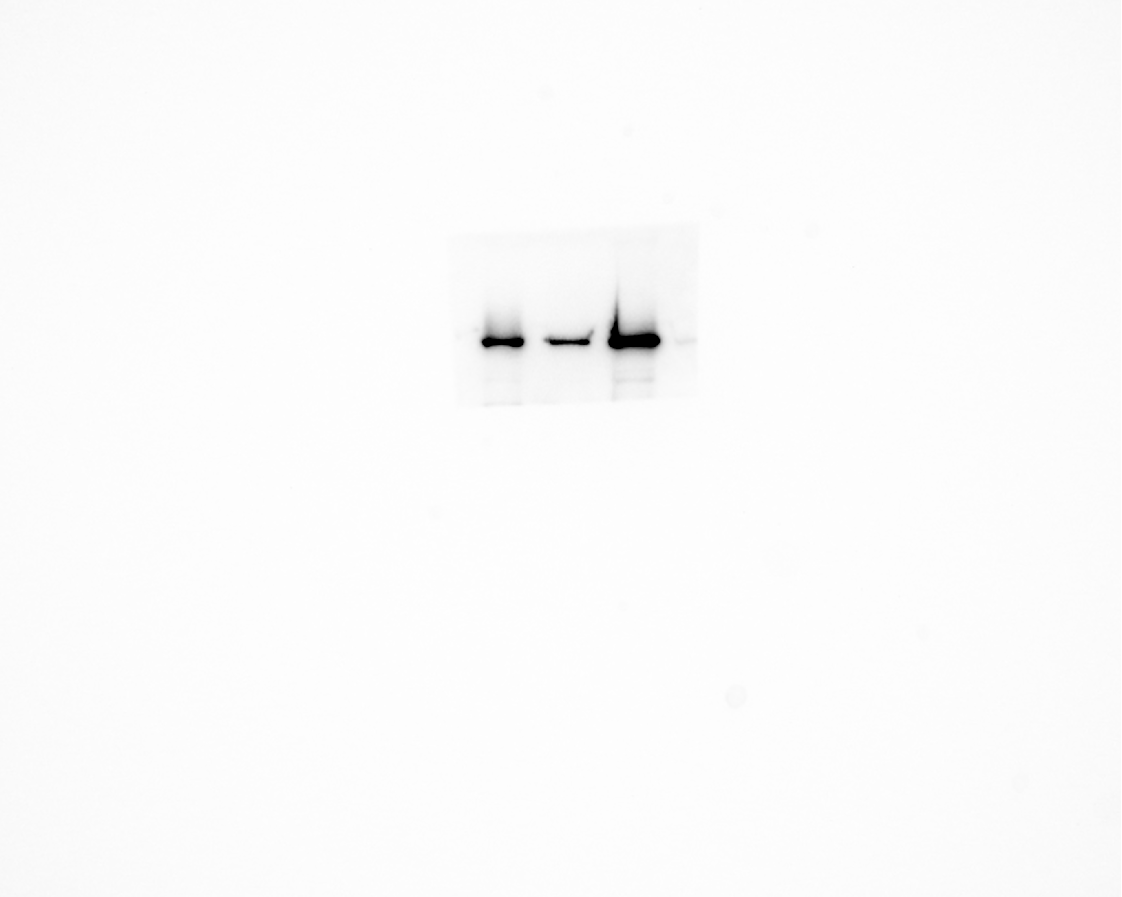


GAPDH


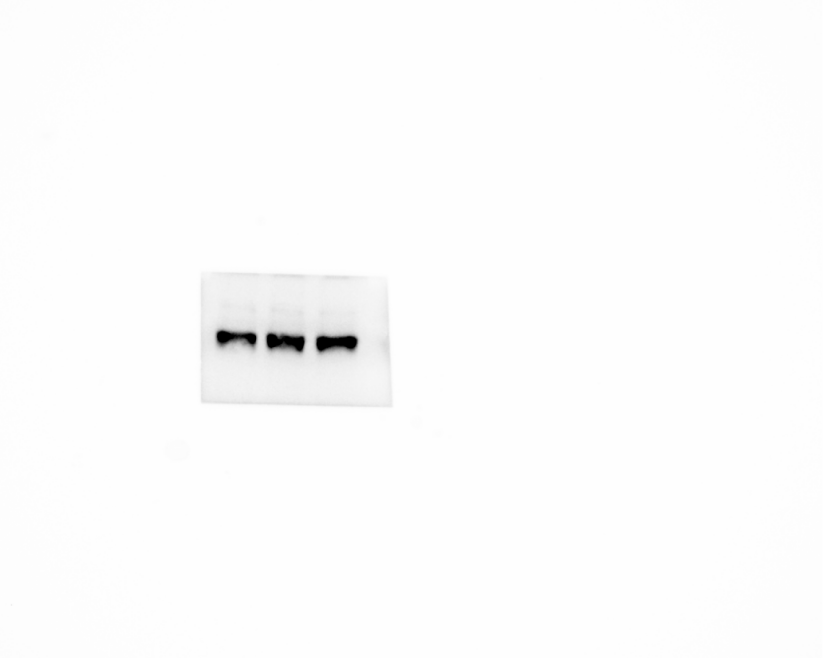

Supplement: Supplementary file 8 — Supplementary Material 8. [file 41065_2025_588_MOESM8_ESM.docx]
